# Supplementary material for: A BLAST from the past: revisiting blastp’s E-value
Source: Bioinformatics. 2024 Dec 4;40(12):btae729. doi: 10.1093/bioinformatics/btae729 (PMC11652269; doi:10.1093/bioinformatics/btae729)
Supplement: btae729_Supplementary_Data [file btae729_supplementary_data.pdf]

Supplement to “A BLAST from the past: revisiting blastp’s E-value”

Yang Lu[1], William Stafford Noble[2,3], and Uri Keich*[4]

[1]Department of Computer Science, University of Waterloo
[2]Department of Genome Sciences, University of Washington
[3]Paul G. Allen School of Computer Science and Engineering, University of Washington
[4]School of Mathematics and Statistics F07, University of Sydney

S1 Supplementary Material

S1.1 Studentizing and location and scale parameters

Claim S1. Suppose S and $Y_1, \ldots, Y_m$ are RVs distributed according to a parametric family $F(\mu, \sigma)$, where μ is a location parameter and σ is a scale parameter. Let $\bar{Y}$ denote the mean and σ_Y denote the standard deviation of the sample $Y_1, \ldots, Y_m$, and let $T = (S - \bar{Y})/\sigma_Y$ be the studentized version of S. Then the distribution of T is invariant of the parameters μ and σ; hence, it can be tabulated even when those parameters are unknown.

Proof.

$$\begin{aligned} T &= \frac{S - \bar{Y}}{\sigma_Y} \\ &= \frac{S - \frac{1}{m}\sum_1^m Y_i}{\sqrt{\frac{1}{m-1}(Y_i - \bar{Y})^2}} \\ &= \frac{(S-\mu) - \frac{1}{m}\sum_1^m (Y_i - \mu)}{\sqrt{\frac{1}{m-1}[(Y_i - \mu) - (\bar{Y} - \mu)]^2}} \\ &= \frac{(S-\mu)/\sigma - \frac{1}{m}\sum_1^m (Y_i - \mu)/\sigma}{\sqrt{\frac{1}{m-1}[(Y_i - \mu)/\sigma - (\bar{Y} - \mu)/\sigma]^2}} \\ &= \frac{\hat{S} - \bar{\hat{Y}}}{\sigma_{\hat{Y}}}, \end{aligned}$$

where $\hat{S} = (S - \mu)/\sigma$ and $\hat{Y}_i = (Y_i - \mu)/\sigma$ are the standardized versions of S and Y_i and hence distributed as $F(0, 1)$. This shows that T can be expressed in terms of $F(0, 1)$ distributed RVs and hence its distribution is invariant of μ and σ. □

*Correspondence: uri.keich@sydney.edu.au

S1.2 The algorithm to compute the SG$_m$ p-values

Algorithm S1: The SG p-value algorithm

Data: A query protein q, a protein database D, a search algorithm $S(q, D)$ that returns a ranked list of alignment scores, a list of observed alignment scores $s = (s_1, \ldots, s_k)$, a specified number of shuffles m, T_p the two-column table of right tail probabilities of SG$_m$ computed in Section 4.1: first column is the bin center, the second is the tail probability.

Result: $PV = (p_1, \ldots, p_k)$ - the list of SG$_m$ p-values of $(s_1, \ldots, s_k)$

```
Y ← [];
for i = 0; i < m; i++ do
    q̃ ← shuffle(q);
    Add Y_i = max(S(q̃, D)) to Y;
end
PV ← [];
Calculate the mean Ȳ and the standard deviation σ_Y of Y;
for j = 0; j < k; j++ do
    Calculate the test statistic t_j = (s_j − Ȳ)/σ_Y;
    if t_j ≤ 0 then
        p_j = 1;
    end
    else if t_j > m_x := max T_p[, 1] then
        /* m_x is the maximal bin recorded in the SG_m table T_p      */
        p_j = T_p[m_x, 2];
        /* The minimum p-value recorded in T_p                        */
    end
    else
        Find the bin i_j in which t_j falls (i.e., T_p[i_j, 1] ≤ t_j ≤ T_p[i_j + 1, 1]) and set p_j = T_p[i_j, 2];
    end
    Add p_j to PV;
end
```

S1.3 Supplementary Table

Search tool	Substitution matrix (Gap open/extension penalty)	The number of reported homologs			
		E-value <0.01	SG_{50} p-value <0.01	E-value <0.05	SG_{50} p-value <0.05
AB-BLAST	BLOSUM62 (11,1)	N/A	138118	N/A	151914
BLAST	BLOSUM62 (11,1)	140488	136921	149846	147360
FASTA	BLOSUM62 (11,1)	128174	133185	139004	144120
AB-BLAST	PAM70 (10,1)	N/A	95183	N/A	104378
BLAST	PAM70 (10,1)	113522	116094	119980	123767
FASTA	PAM70 (10,1)	88706	91648	97284	100848
AB-BLAST	PFASUM60 (15,1)	N/A	146834	N/A	159705
FASTA	PFASUM60 (15,1)	133351	137729	144578	148840
SSEARCH	PFASUM60 (15,1)	145288	149736	157231	162083

Table S1: **The number of ASTRAL40 homologous sequences reported at the given threshold**. See Section 4.4.2 for details.

S1.4 Supplementary Figures

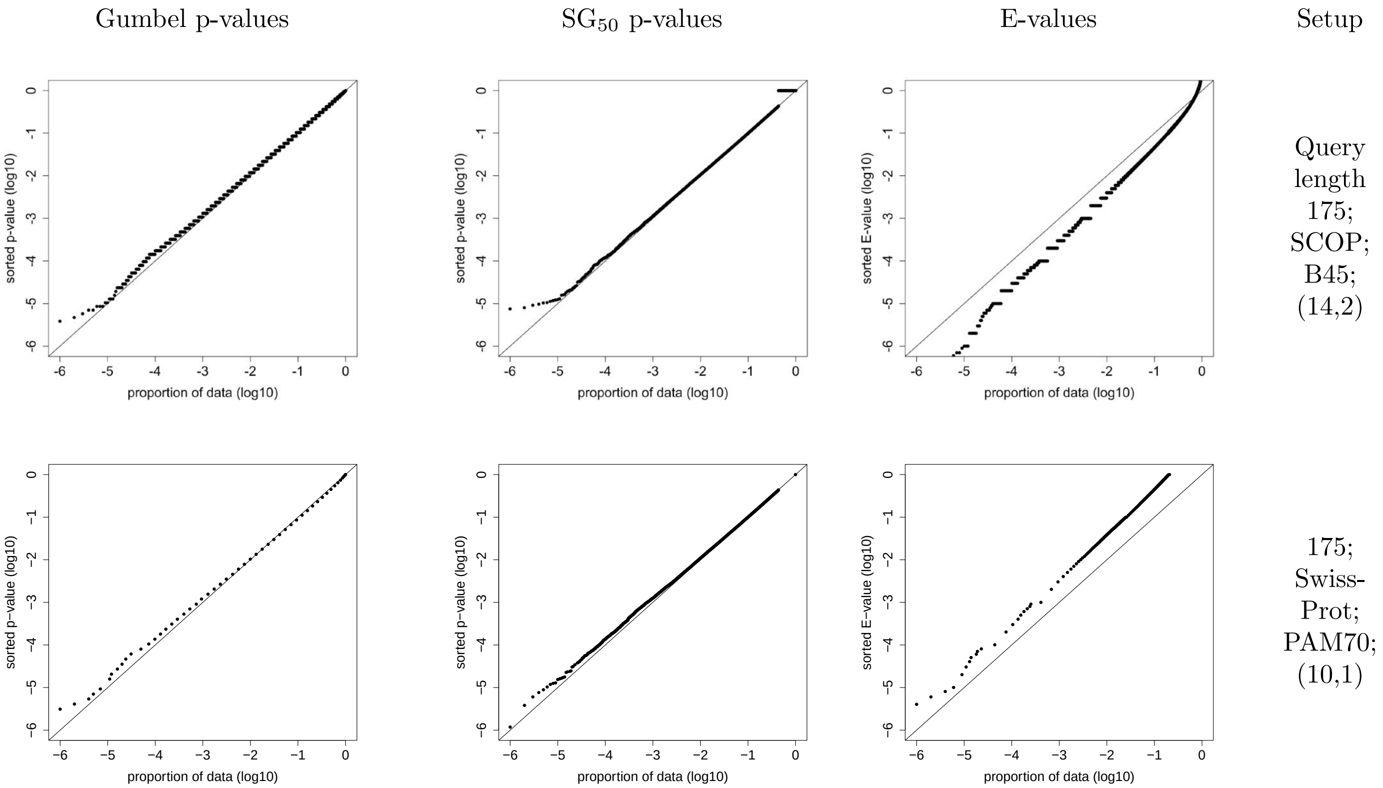

Figure S1: **BLAST E-value can be liberal as well as conservative**. The top row plots were made based on a sample generated by using BLAST to search 10^6 shuffles of a query of length 175 amino acids against the SCOP database with BLOSUM45 and its BLAST default gap penalties of (14,2). The bottom row is similarly based on 10^6 BLAST searches against the Swiss-Prot database with PAM70 and its BLAST default gap penalties of (10,1). The left panel examines the fit of the optimal alignment score to the Gumbel, the middle one the validity of our SG_{50} p-values, and the right panel examines the validity of the E-values (see Section 4.4.1 for details).

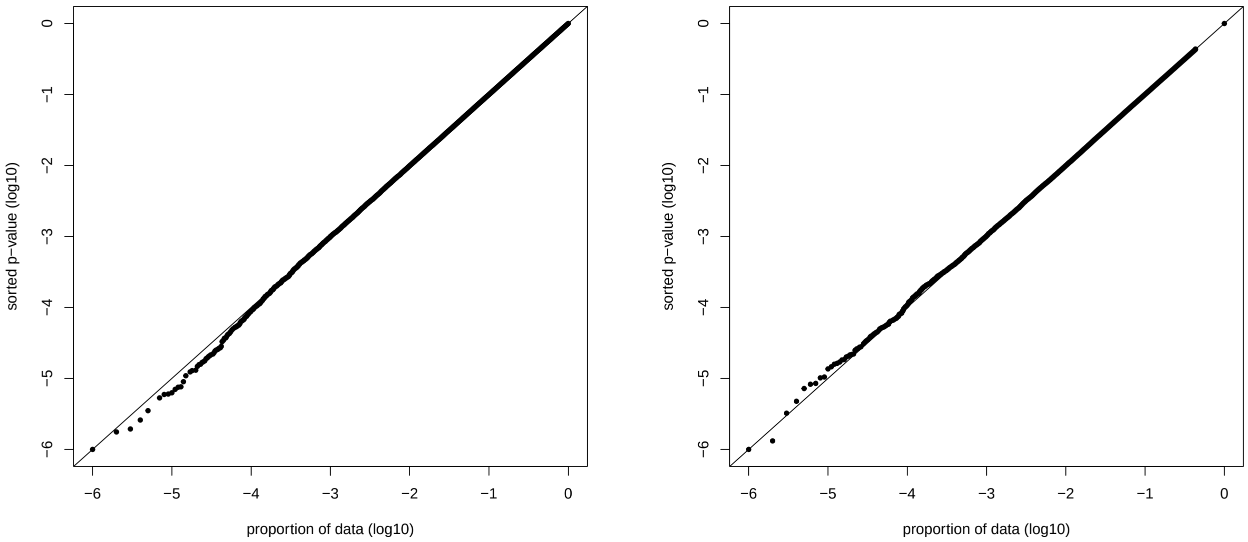

A: Gumbel p-values applied to a Gumbel sample **B: SG_{50} p-values of a Gumbel sample**

Figure S2: **When the sample is drawn from the Gumbel distribution**. A sample of size 10^6 was taken from the standard Gumbel distribution in order to provide a reference to our probability plots when the sample is drawn from the exact distribution rather than an approximated Gumbel. (A): The probability plot comparing the sample and the CDF of the sample's MLE fitted Gumbel. Specifically, the y-axis denotes the (log10 of the) right tail probability (p-value) of the sample points computed using the sample-fitted Gumbel. The points were sorted in decreasing order and the x-axis denotes the (log10 of the) fraction of sample points that are smaller. (B): Similar to (A) only the p-value of each sample point is the SG_{50} p-value computed using the tail probability table described in Section 4.1. Specifically, each Gumbel sample value from (A) was studentized using an independent sample of size $m = 50$ from the standard Gumbel distribution, and the corresponding tail probability of the studentized value was taken from the table.

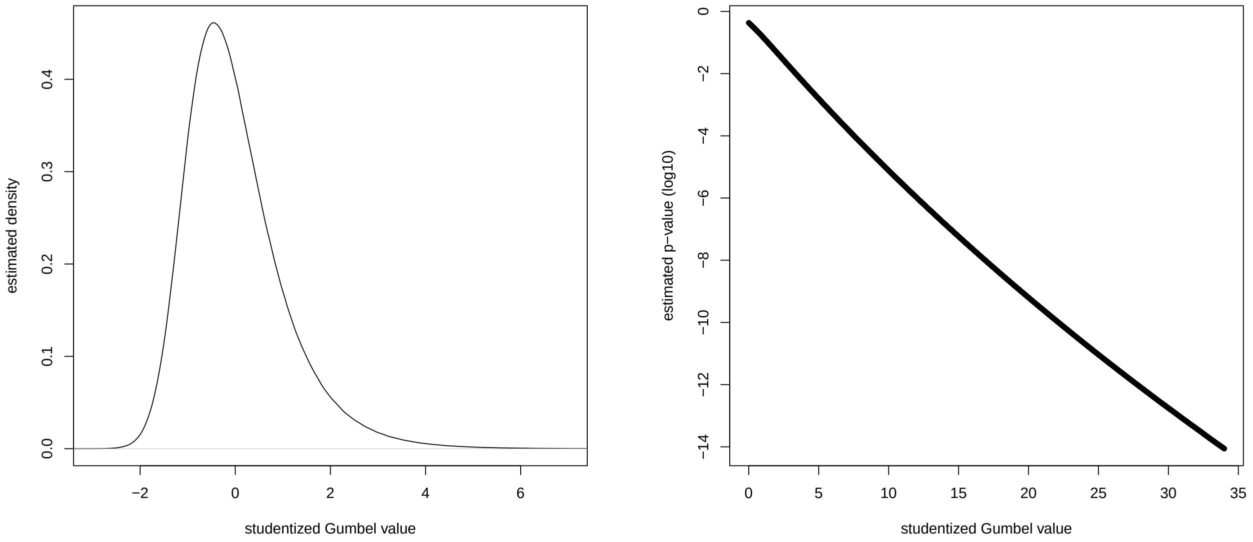

A: Estimated SG_{50} density **B: The range of SG_{50} p-values we pre-computed**

Figure S3: **The SG_{50} distribution**. (A): A sample of size 10^7 was taken from the SG_{50} distribution and a density was estimated using the `R` function `density`. (B): The log10 of the right tail probabilities of the SG_{50} distribution estimated via importance sampling as described in Section 4.1.

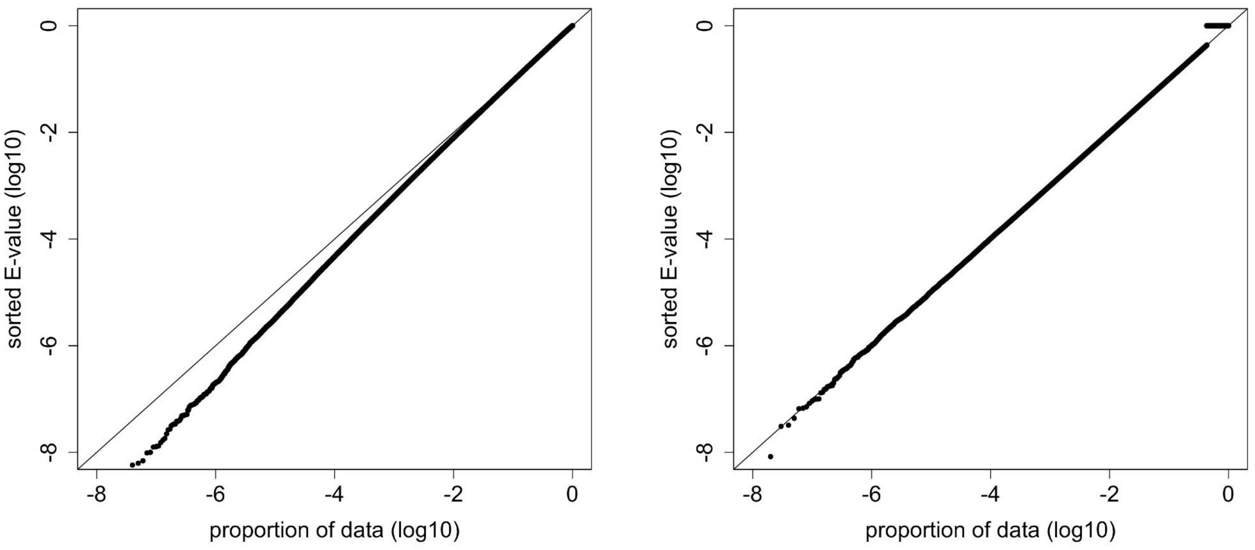

A: MLE p-values **B: SG_{50} p-values**

Figure S4: **The MLE p-values are not valid**. (A): A sample of size 10^8 of Gumbel MLE p-values was generated by sampling 10^8 independent sets of $1 + m$ standard Gumbel samples: $(s_i, y_{i1}, y_{i2}, \ldots, y_{im})$ for $i = 1, 2, \ldots, 10^8$ ($m = 50$ here). Then $y_{i1}, \ldots, y_{im}$ were used to find the MLE of the Gumbel rate and location parameters, which were plugged into the Gumbel CDF to estimate the p-value of s_i. The probability plot above shows these MLE p-values are invalid. Specifically, for example, a p-value of 10^{-5} or smaller was observed at a rate of $2.6 \cdot 10{-}5$, i.e., 2.6 times more often than expected (binomial test p-value of 0). (B): The same 10^8 sampled sets that were used in (A) were used here to compute the SG_{50} p-value of each s_i. Specifically, instead of estimating the Gumbel parameters from the $y_{i\bullet}$ sample, it was used to studentize s_i and the p-value was taken from our tabulated right tail of SG_{50}. The SG_{50} p-values of the same sample used in (A) appear to be valid.

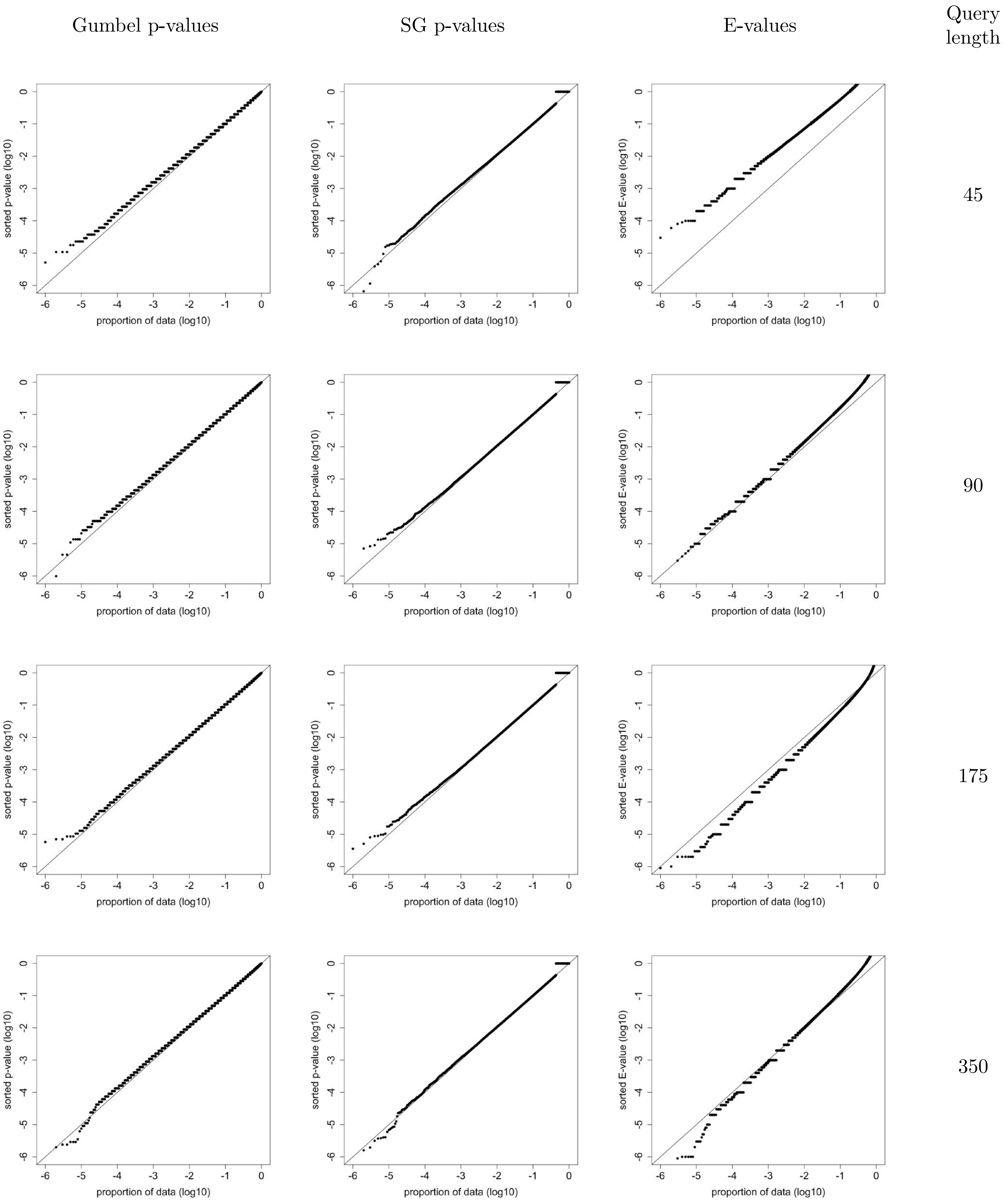

Figure S5: **Using BLAST to search 10^6 shuffled queries against the Swiss-Prot database with BLOSUM45 (14,2)**. Five domains from the SCOP database of lengths 45, 90, 175, and 350 were selected, and 10^6 shuffles of each were blasted against Swiss-Prot using BLOSUM45 (14,2) and the database-wide optimal local alignment (raw) score and E-values were noted. Each of the probability plots specifies the (log10 of the) fraction of the shuffles (x-axis) for which the optimal alignment p-value/E-value is better than the (log10 of the) reported optimal p-value/E-value (y-axis). Note that the y-axis was constrained to match the range of the x-axis (log10 of the number of shuffles). BLAST's E-values here are at times overly conservative, e.g., only 0.1% of the length 45 E-values are ≤ 0.01, and at others too liberal, e.g., 7.7% of the length 175 E-values are ≤ 0.05 (binomial test p-value of 0). In contrast, the SG_{50} p-values are valid and better calibrated for all query lengths.

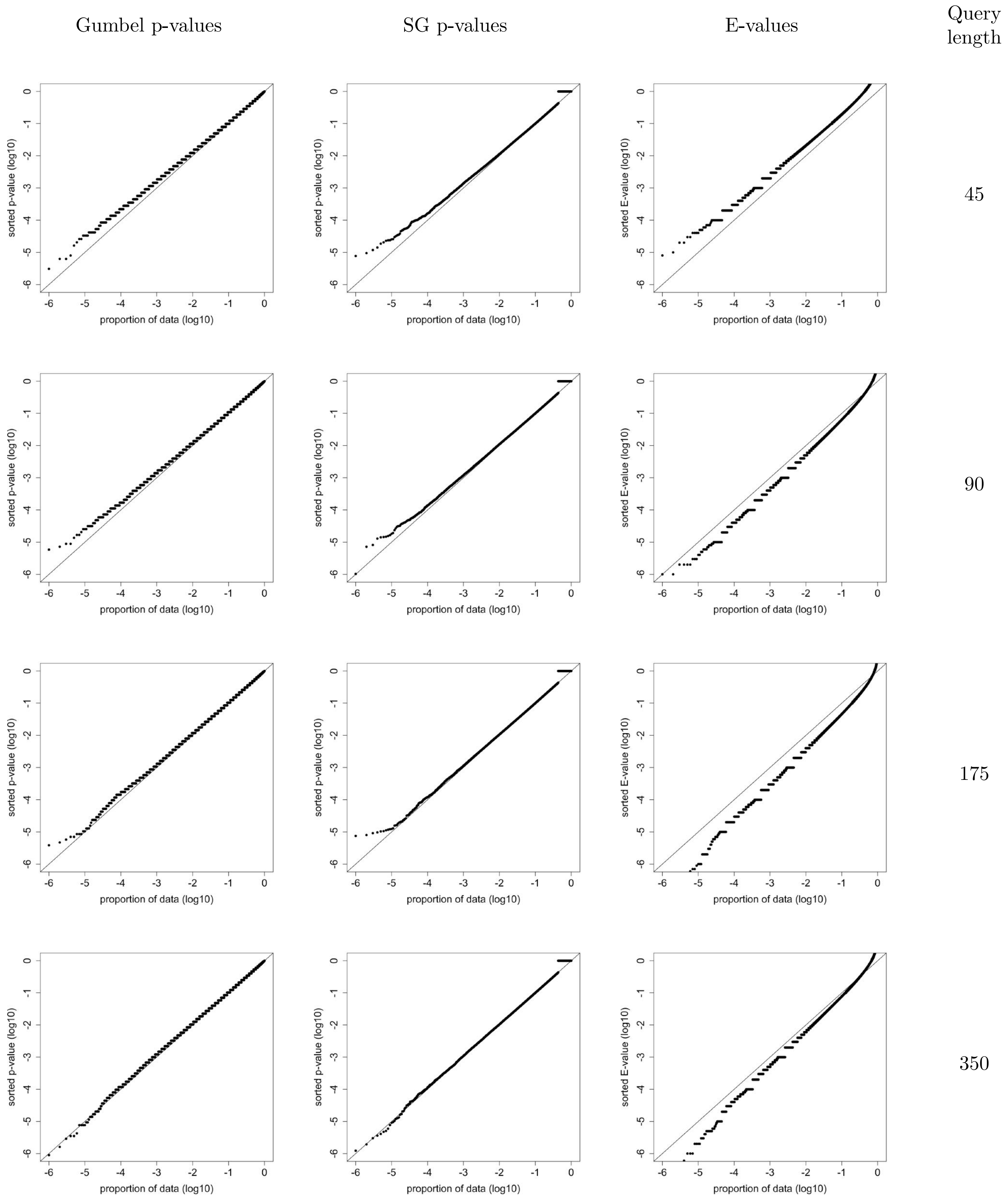

Figure S6: **Using BLAST to search 10^6 shuffled queries against the SCOP database with BLOSUM45 (14,2)**. Same as Figure S5 only searching the SCOP database (rather than Swiss-Prot). Note that the y-axis was constrained to match the range of the x-axis (log10 of the number of shuffles). While not as conservative as when searching the length 45 queries Swiss-Prot, searching SCOP with the same BLOSUM45 (14,2) is visibly more liberally biased, e.g., for length 175 11% of the length 175 E-values are ≤ 0.05 (p-value of 0). In contrast, the SG_{50} p-values are valid and better calibrated for all query lengths.

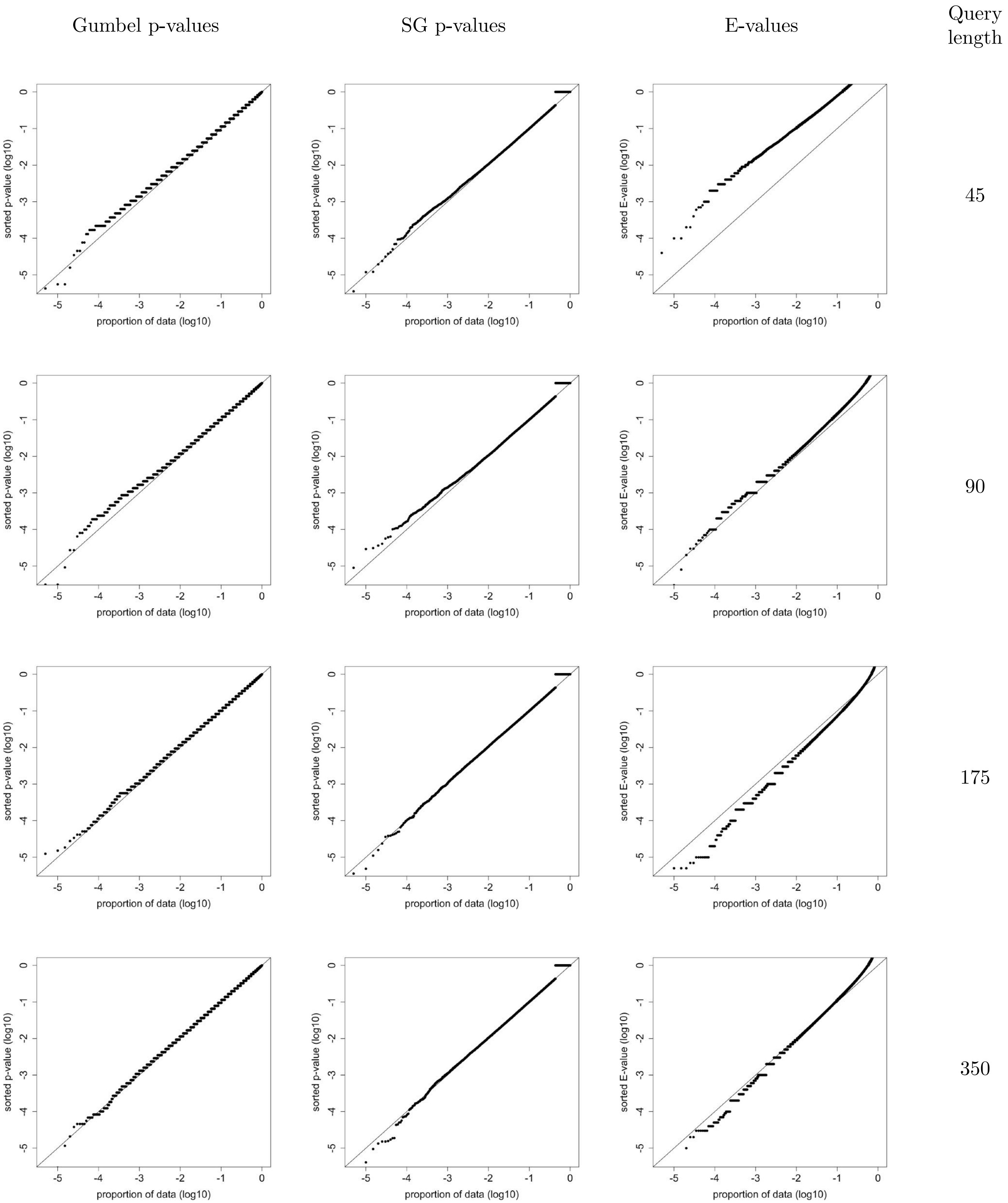

Figure S7: **Using BLAST to search 200,000 shuffled *iid-sampled* queries against the Swiss-Prot database using BLOSUM45 (14,2)**. Similar to Figure S5 but blasting (against Swiss-Prot using BLOSUM45 (14,2)) 200,000 random shuffles of five queries of length 45, 90, 175 and 350 residues, which were randomly drawn according to an iid process. Note that the y-axis was constrained to match the range of the x-axis (log10 of the number of shuffles). Qualitatively the results are the same as when searching the same Swiss-Prot using the 10^6 shuffles of the four selected SCOP domains (Figure S5).

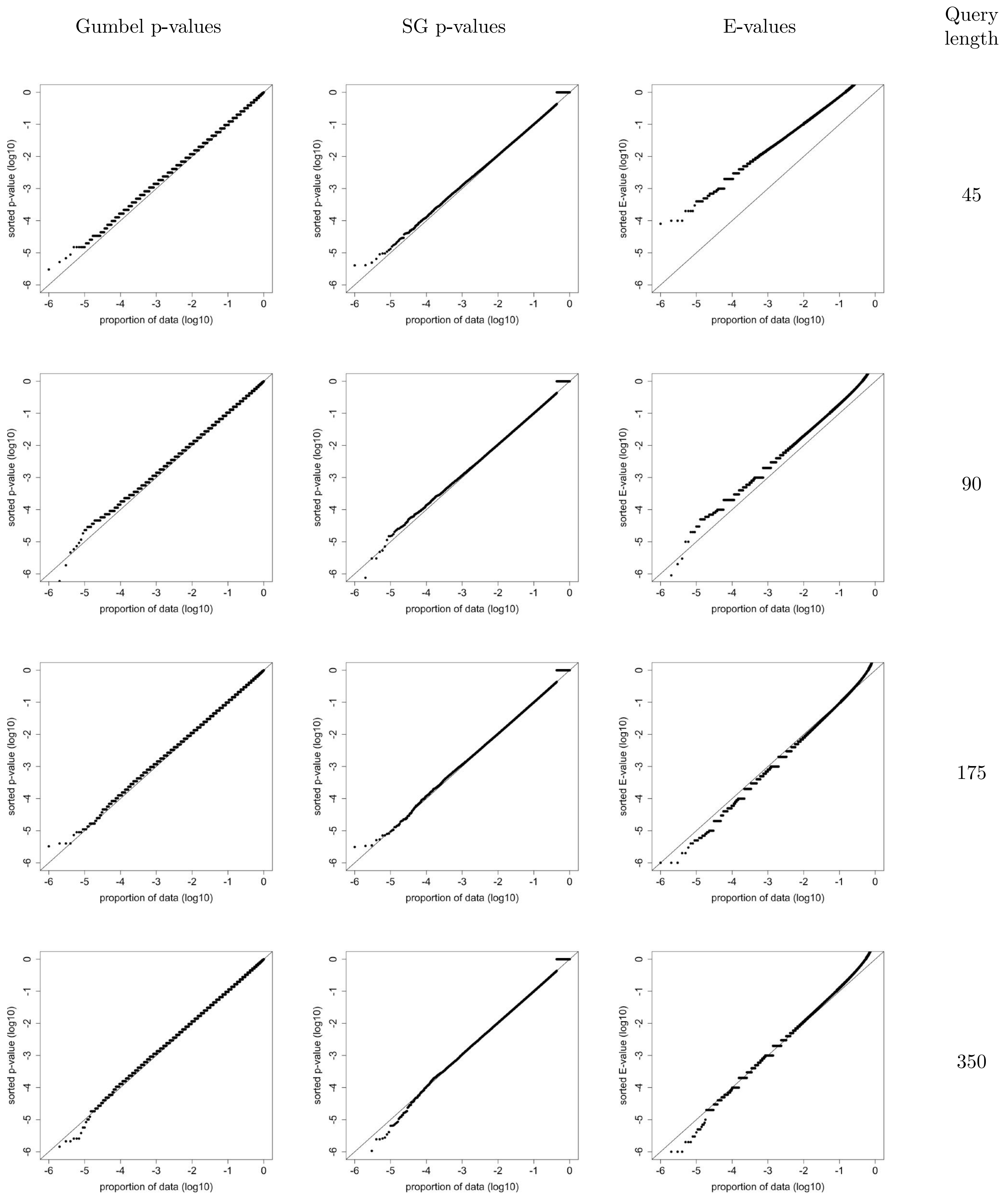

Figure S8: **Using BLAST to search 10^6 shuffled queries against the Swiss-Prot database with BLOSUM50 (13,2)**. Same as Figure S5 only searching using BLOSUM50 (13,2) (instead of BLOSUM45). The E-values here are at times overly conservatives, e.g., only 0.06% of the length 45 E-values are ≤ 0.01, and at others too liberal, e.g., 5.7% of the length 175 E-values are ≤ 0.05 (binomial test p-value of $5 \cdot 10^{-197}$). In contrast, the SG_{50} p-values are valid and better calibrated for all query lengths.

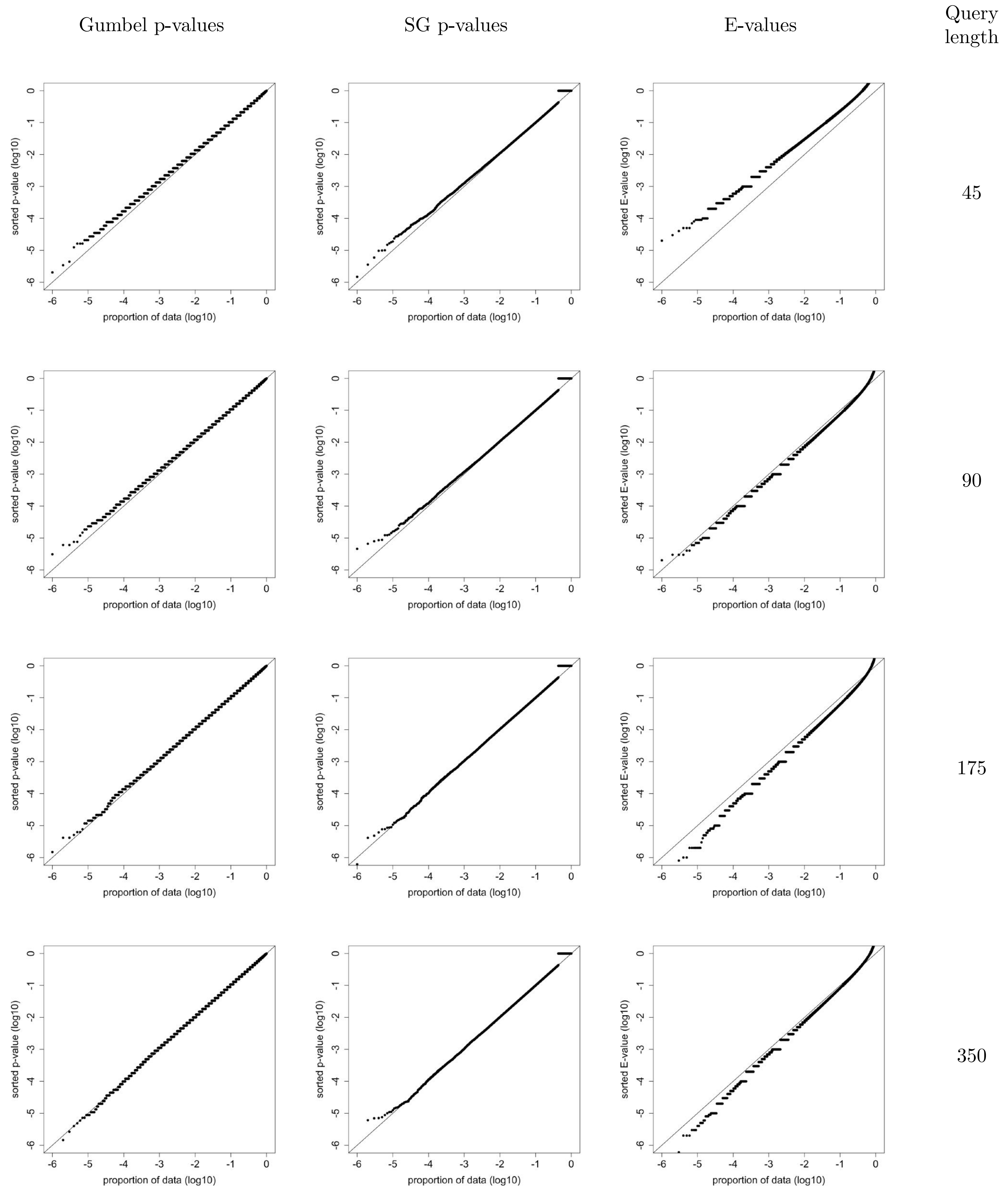

Figure S9: **Using BLAST to search 10^6 shuffled queries against the SCOP database with BLOSUM50 (13,2)**. Same as Figure S6 except searching using BLOSUM50 (13,2). For the length 175 queries there are more than 3 times as many E-values ≤ 0.001 as expected (p-value of 0).

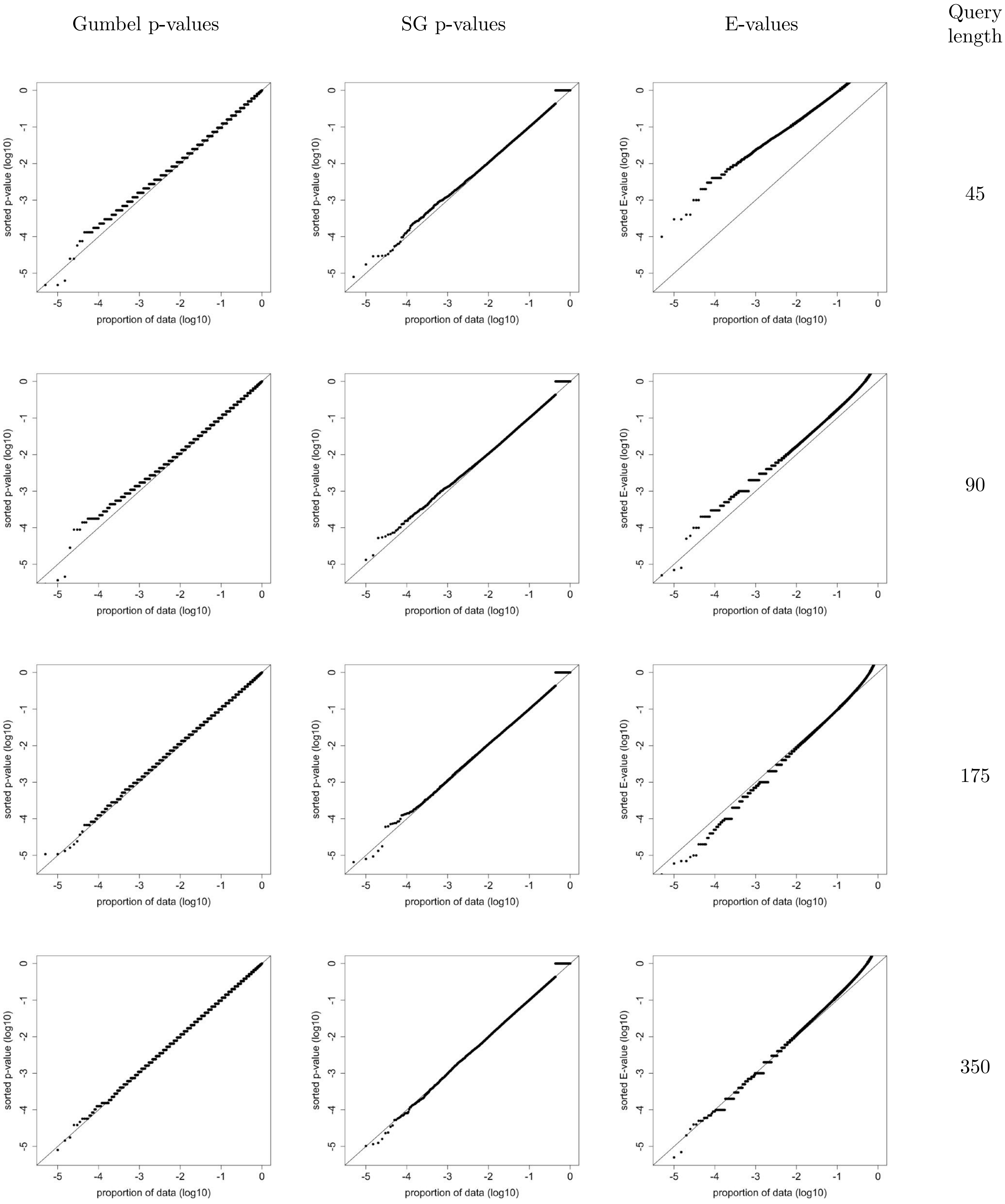

Figure S10: **Using BLAST to search 200,000 shuffled *iid-sampled* queries against the Swiss-Prot database using BLOSUM50 (13,2)**. Same as Figure S7 only using BLOSUM50 (13,2).

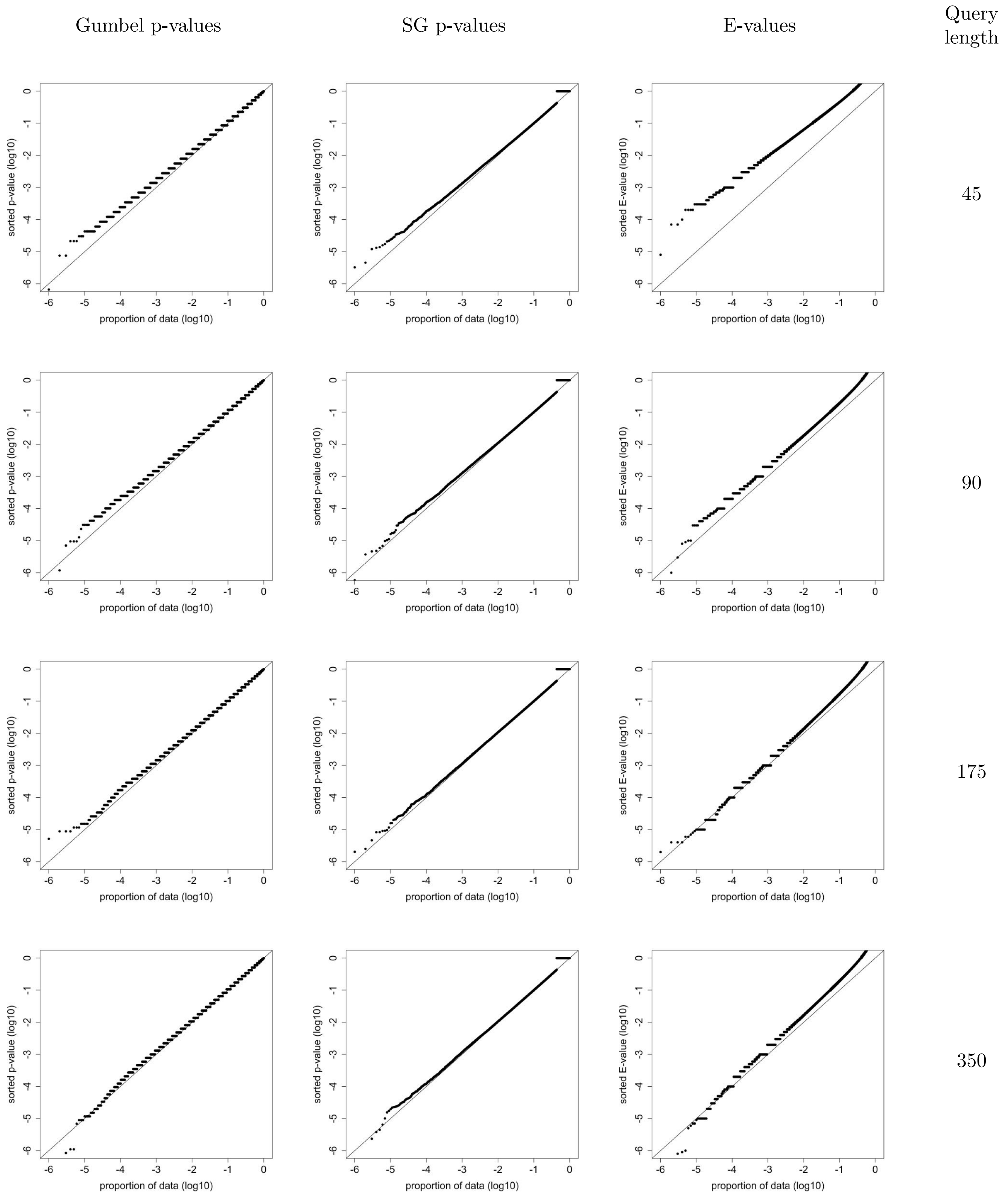

Figure S11: **Using BLAST to search 10^6 shuffled queries against the Swiss-Prot database with BLOSUM62 (11,1)**. Same as Figure S5 except searching using BLOSUM62 (11,1). Only 4 of the length 45 shuffles and 6 of the length 90 shuffles have an E-value $\leq 10^{-4}$ instead of the expected 100 in each case. At the same time there 22% more length 175 E-values that are ≤ 0.001 than expected (p-value of $1.7 \cdot 10^{-11}$).

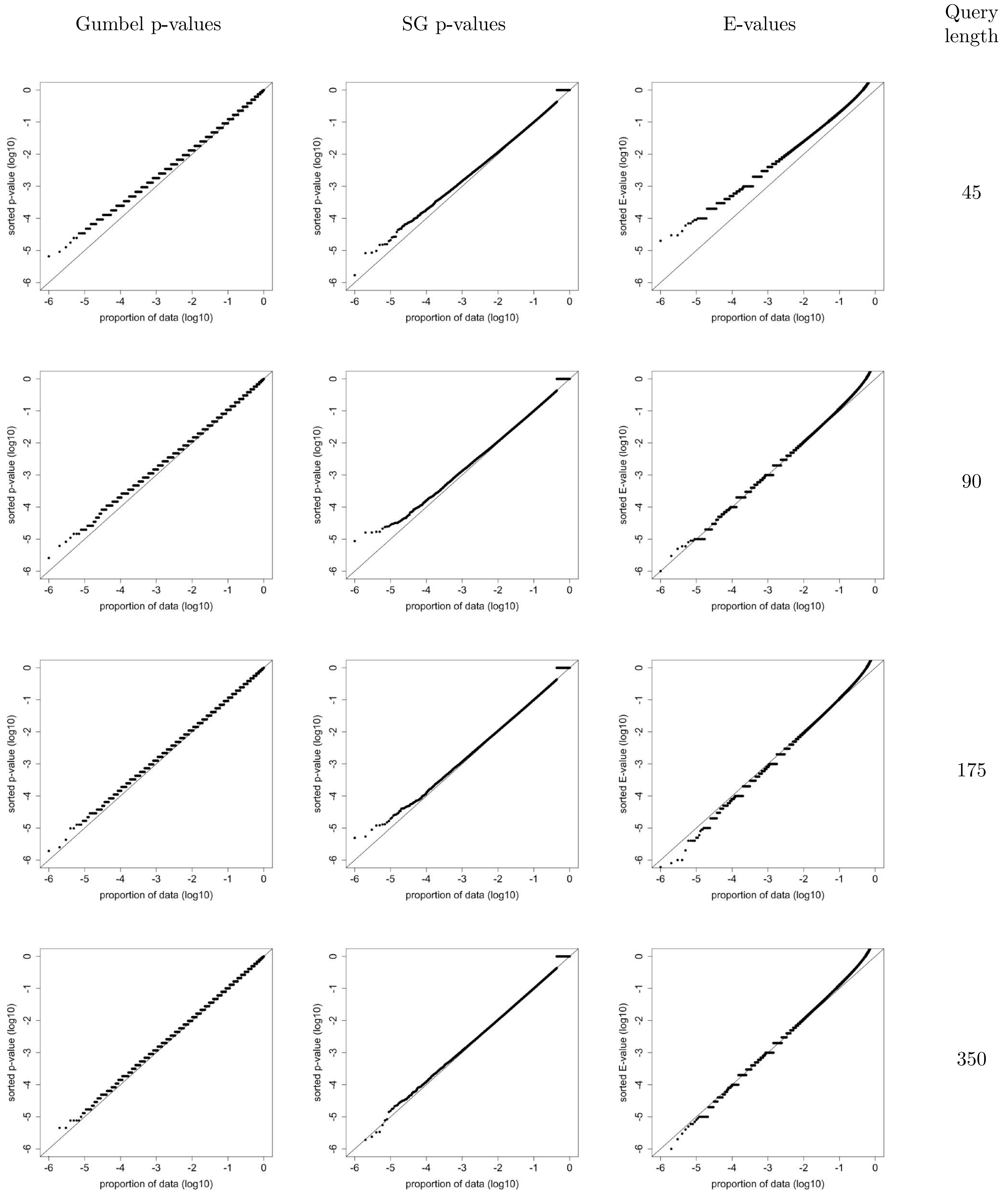

Figure S12: **Using BLAST to search 10^6 shuffled queries against the SCOP database with BLOSUM62 (11,1)**. Same as Figure S6 except searching using BLOSUM62 (11,1). For the length 175 shuffles there are 78% more E-values ≤ 0.001 than expected (p-value of $5 \cdot 10^{-109}$).

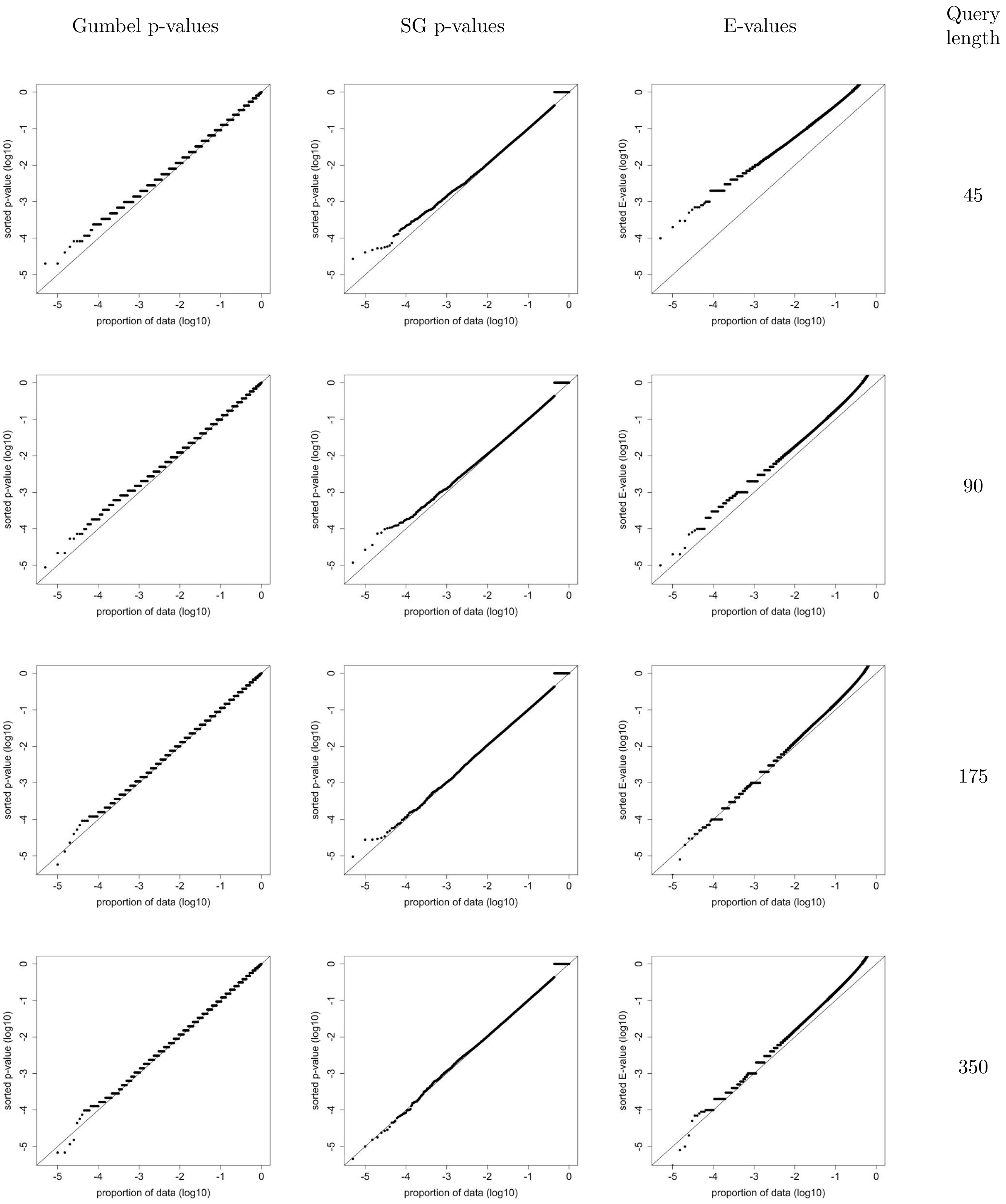

Figure S13: **Using BLAST to search 200,000 shuffled *iid-sampled* queries against the Swiss-Prot database using BLOSUM62 (11,1)**. Same as Figure S7 only using BLOSUM62 (11,1).

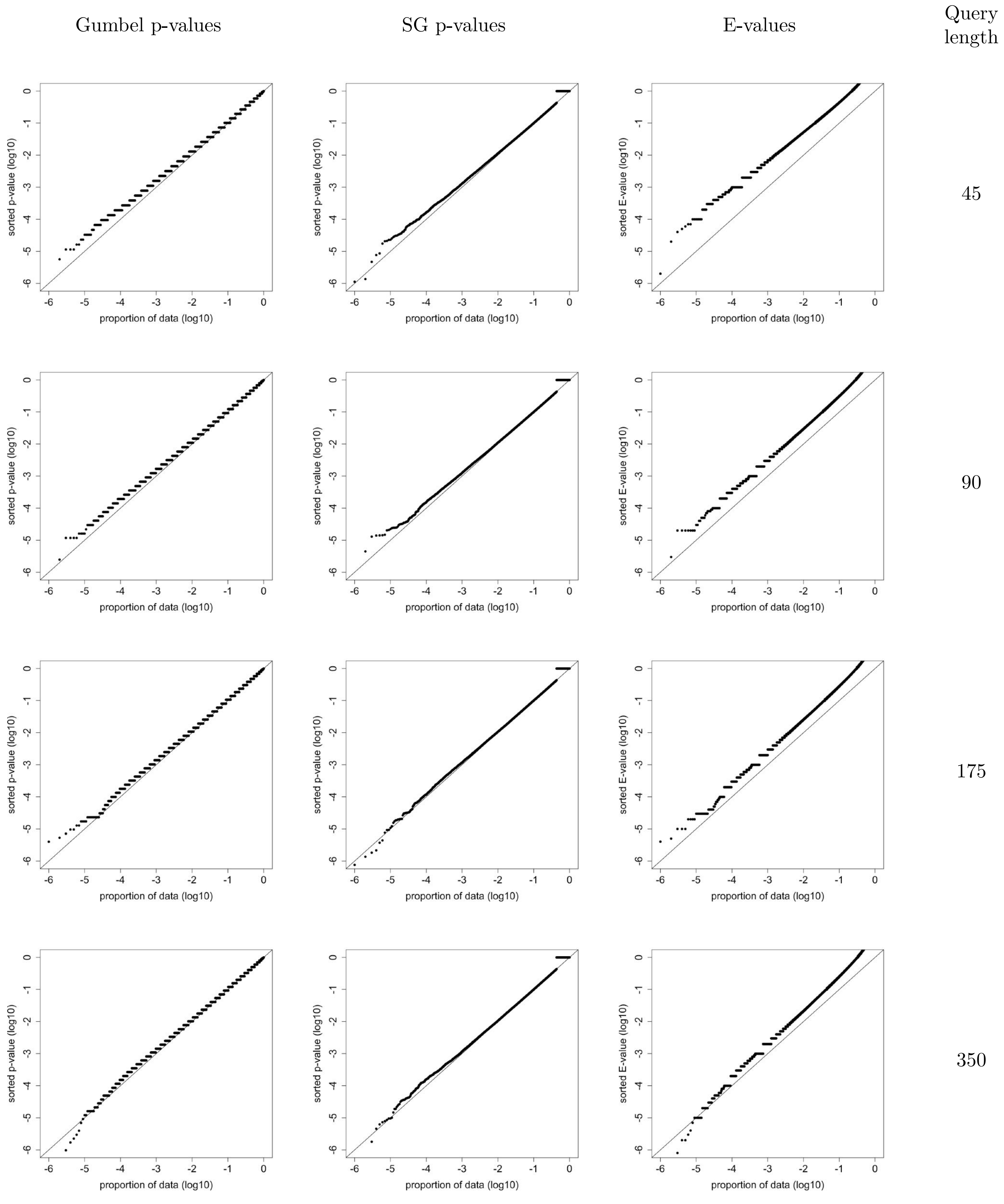

Figure S14: **Using BLAST to search 10^6 shuffled queries against the Swiss-Prot database with BLOSUM80 (10,1)**. Same as Figure S5 except searching using BLOSUM80.

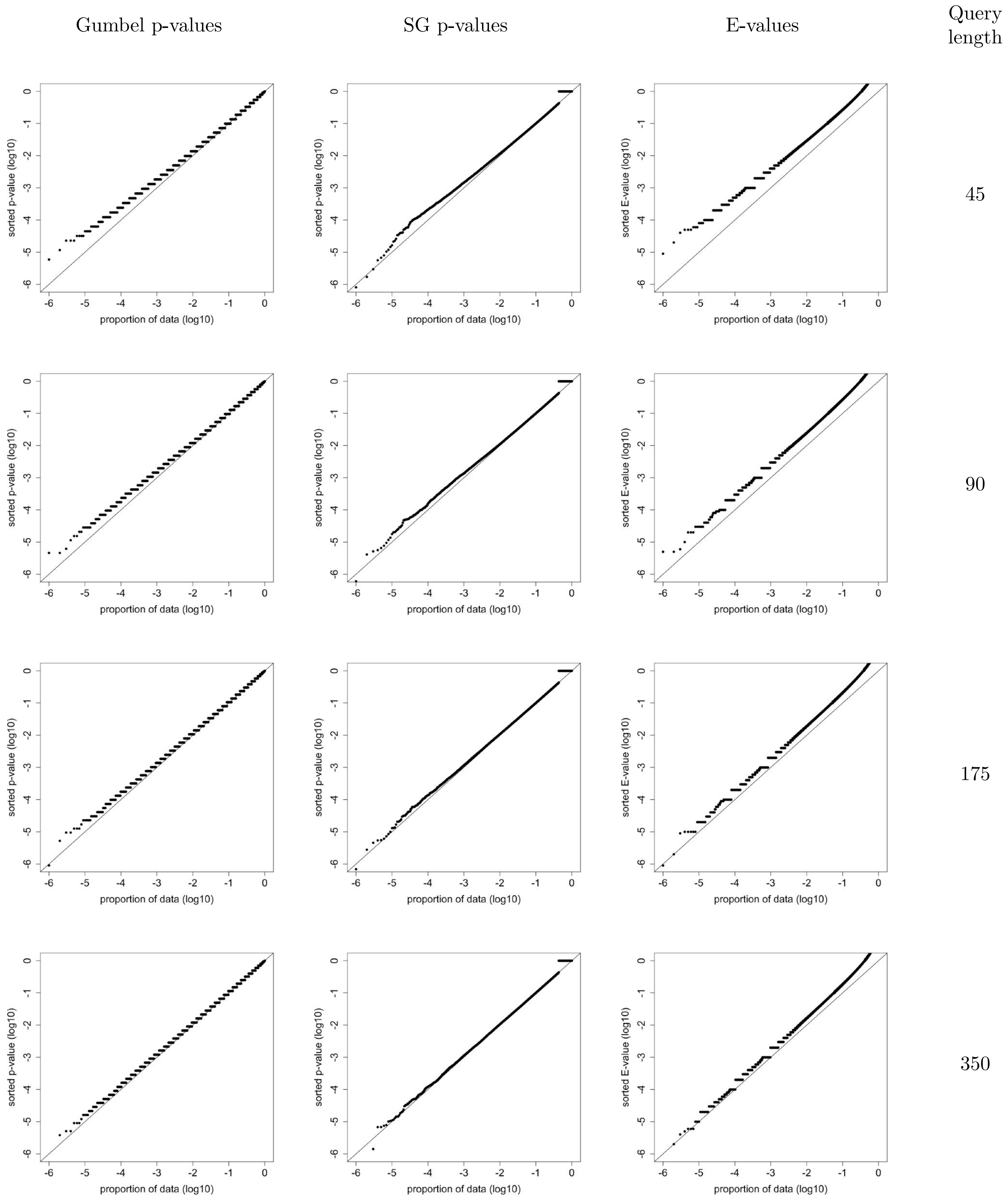

Figure S15: **Using BLAST to search 10^6 shuffled queries against the SCOP database with BLOSUM80 (10,1)**. Same as Figure S6 except searching using BLOSUM80 (10,1).

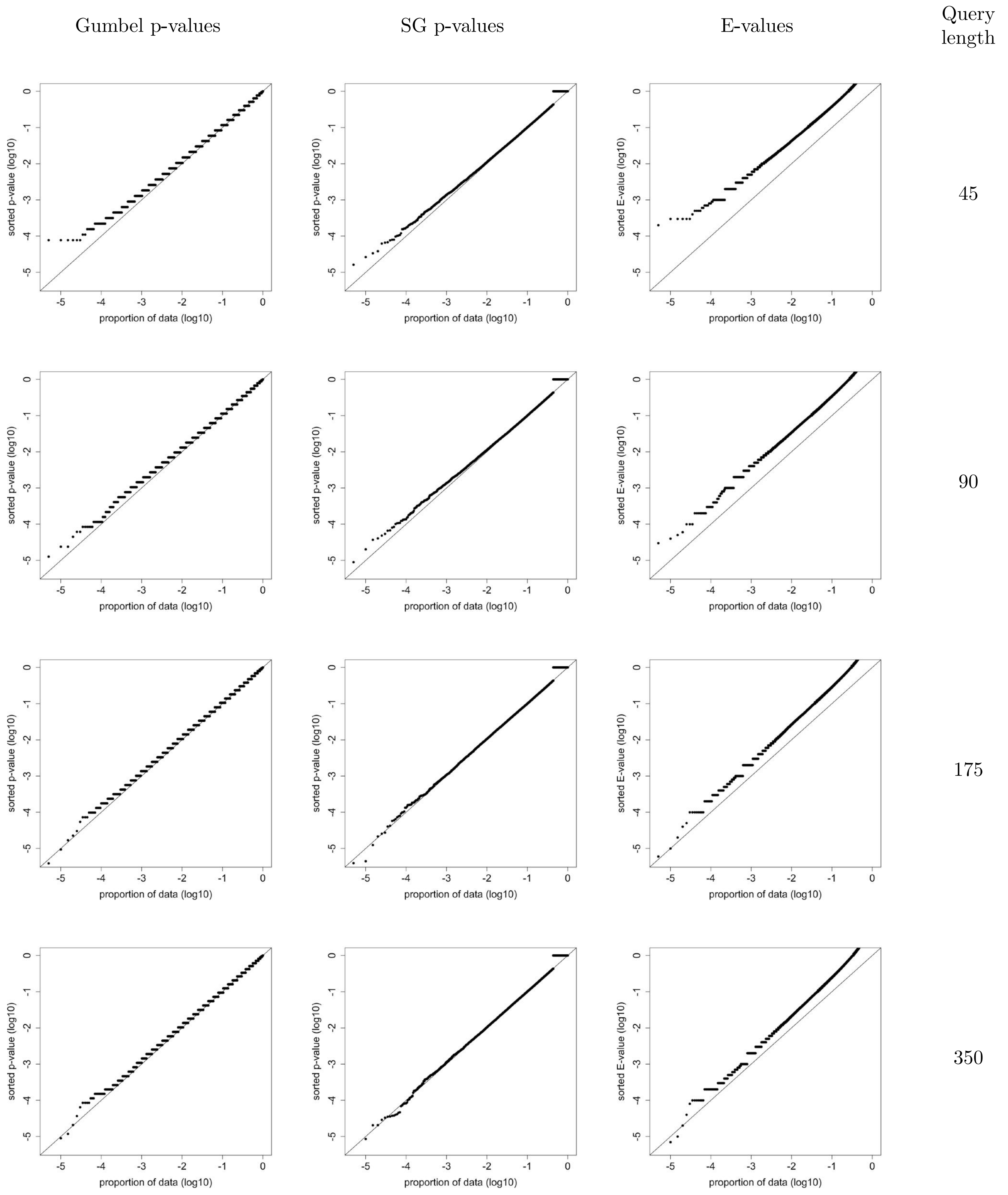

Figure S16: **Using BLAST to search 200,000 shuffled *iid-sampled* queries against the Swiss-Prot database using BLOSUM80 (10,1)**. Same as Figure S7 except using BLOSUM80 (10,1).

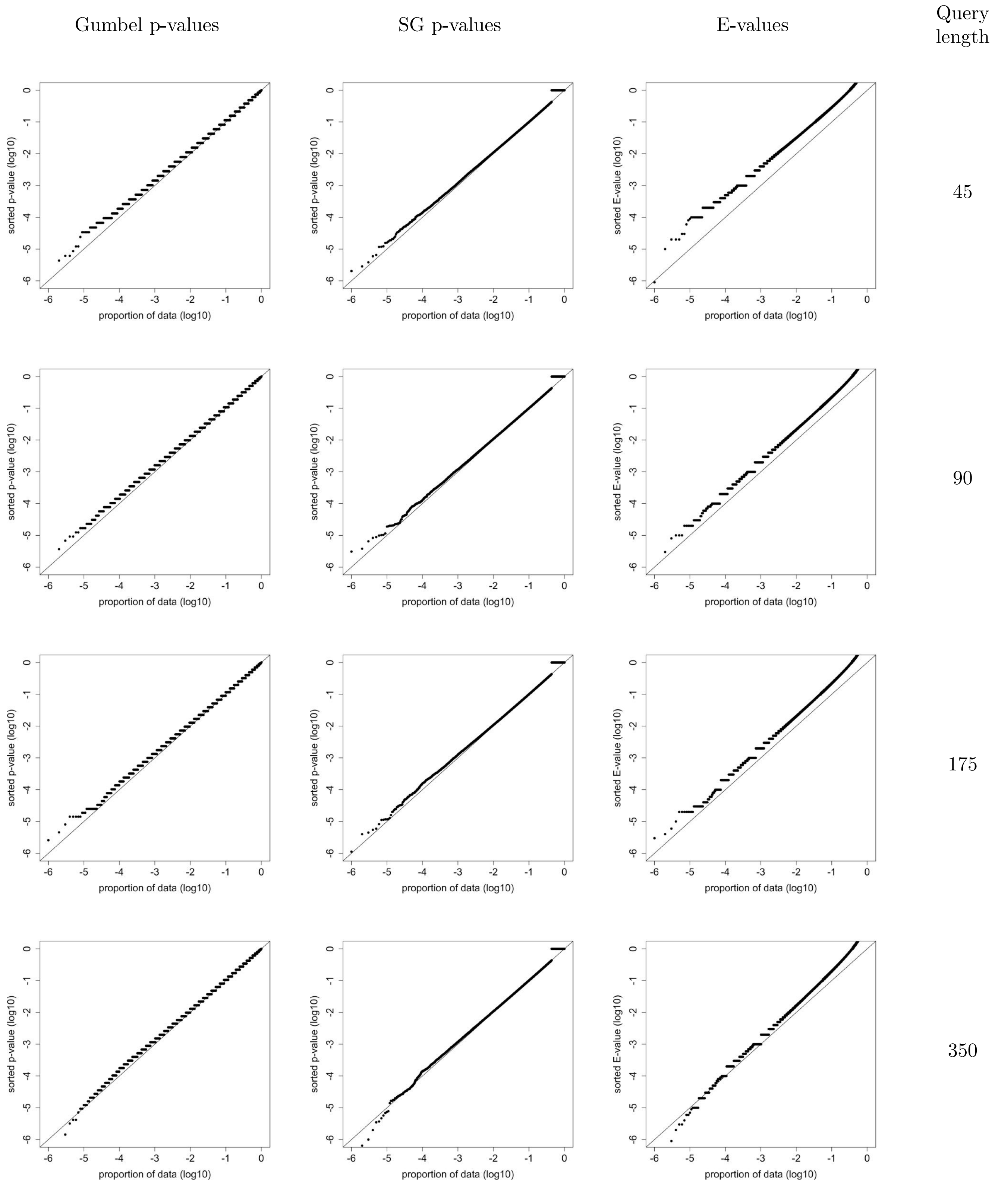

Figure S17: **Using BLAST to search 10^6 shuffled queries against the Swiss-Prot database with BLOSUM90 (10,1)**. Same as Figure S5 except searching using BLOSUM90.

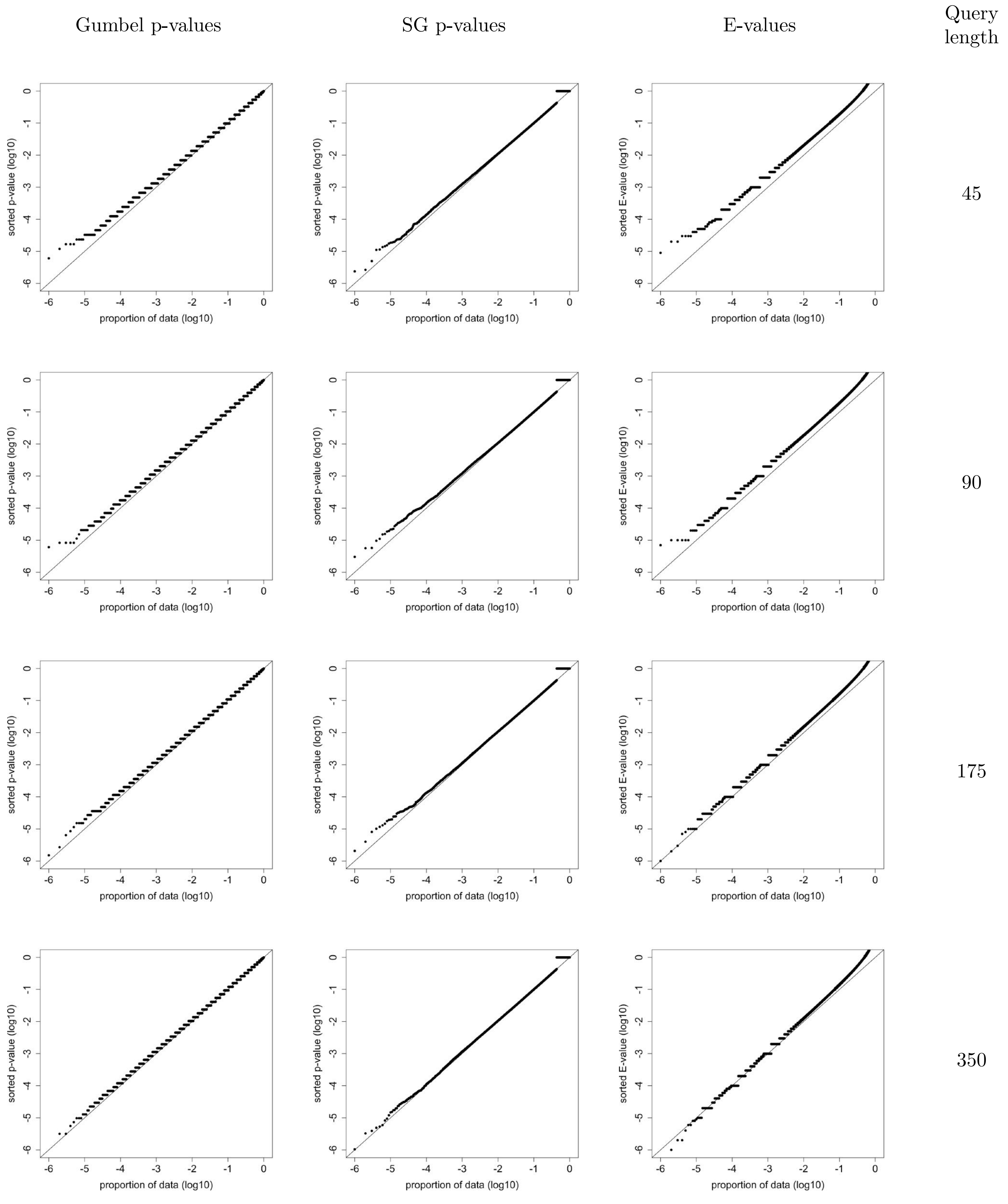

Figure S18: **Using BLAST to search 10^6 shuffled queries against the SCOP database with BLOSUM90 (10,1)**. Same as Figure S6 except searching using BLOSUM90 (10,1).

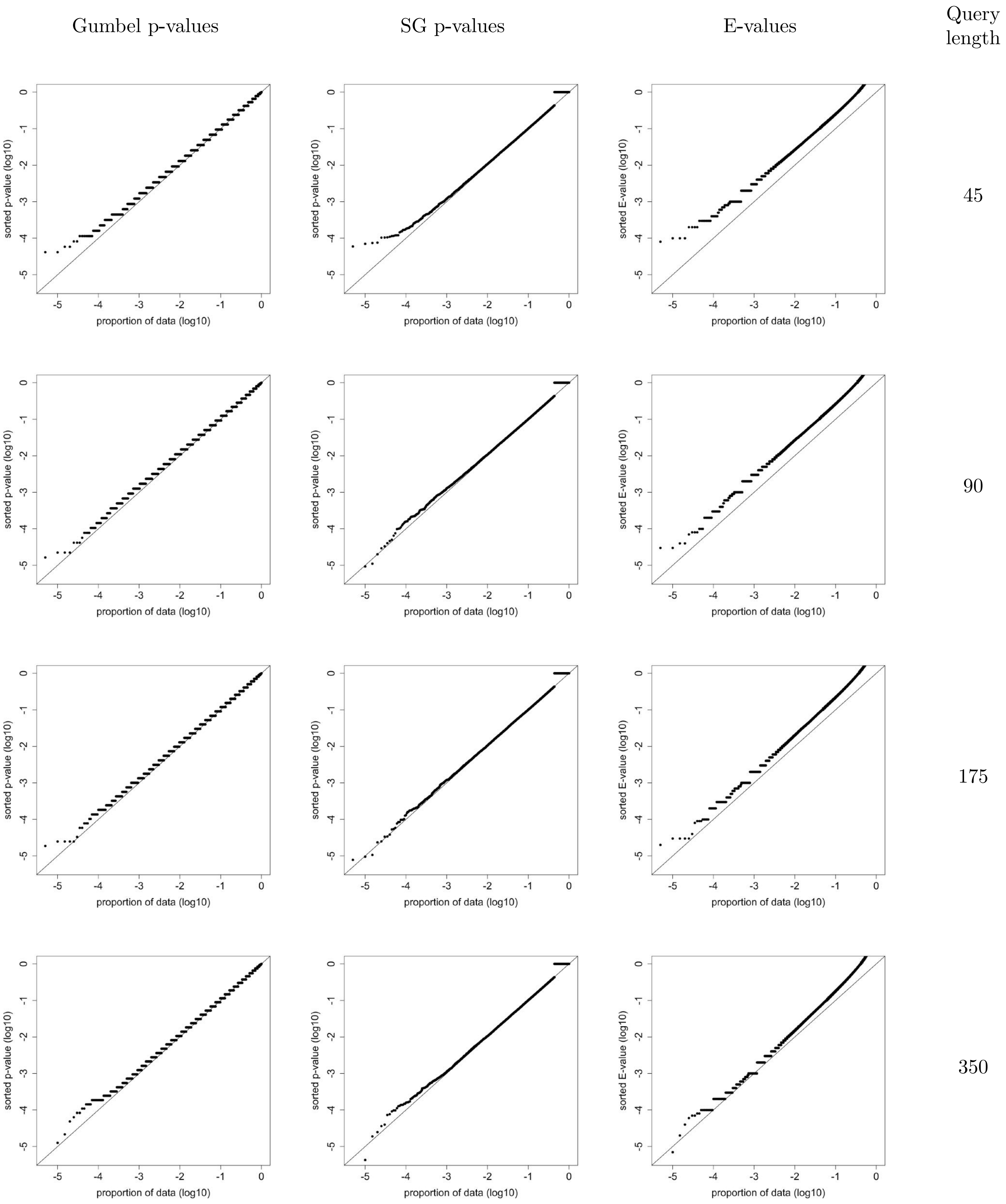

Figure S19: **Using BLAST to search 200,000 shuffled *iid-sampled* queries against the Swiss-Prot database using BLOSUM90 (10,1).** Same as Figure S7 except using BLOSUM90 (10,1).

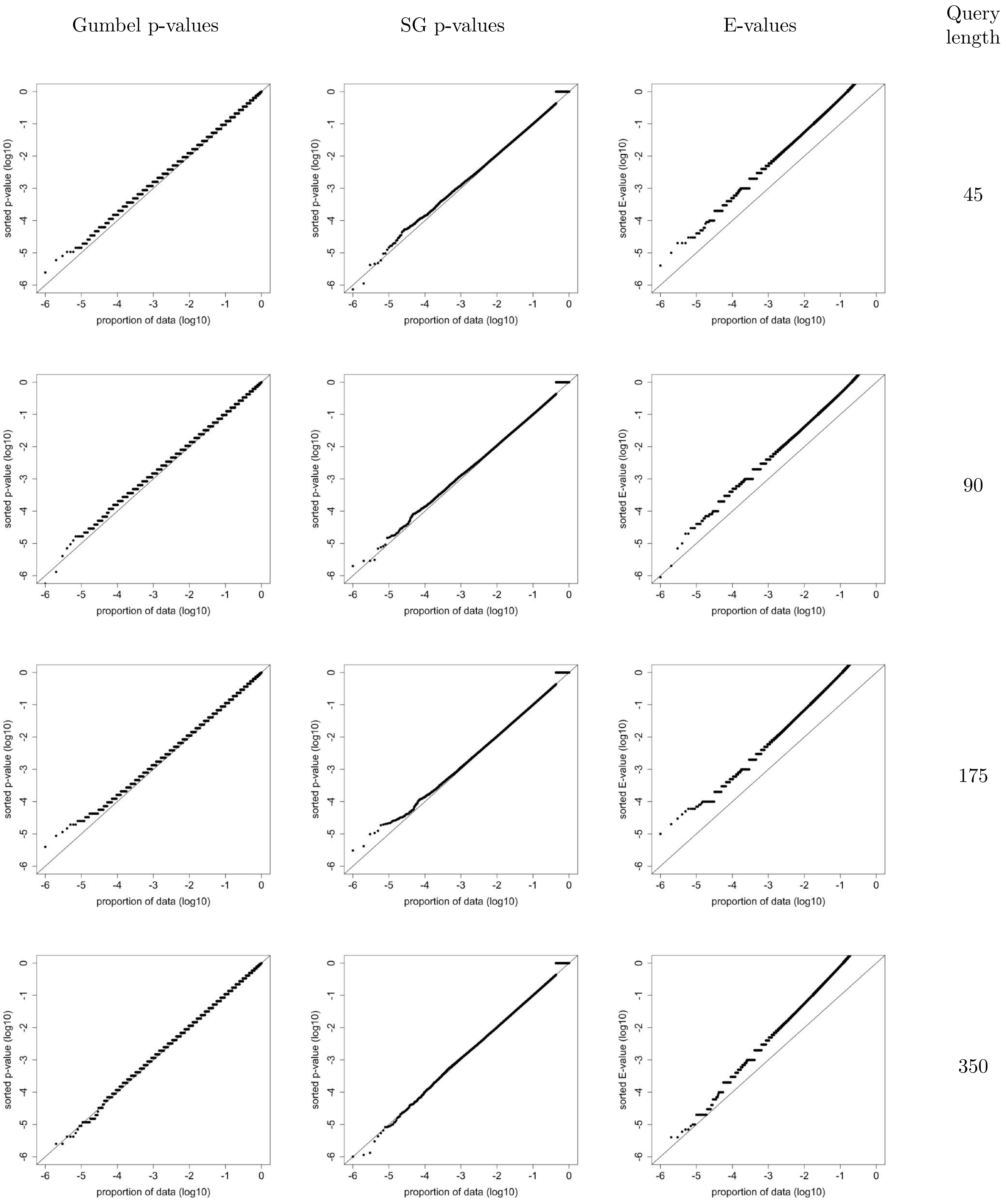

Figure S20: **Using BLAST to search 10^6 shuffled queries against the Swiss-Prot database with PAM30 (9,1).** Same as Figure S5 except searching using PAM30 (9,1).

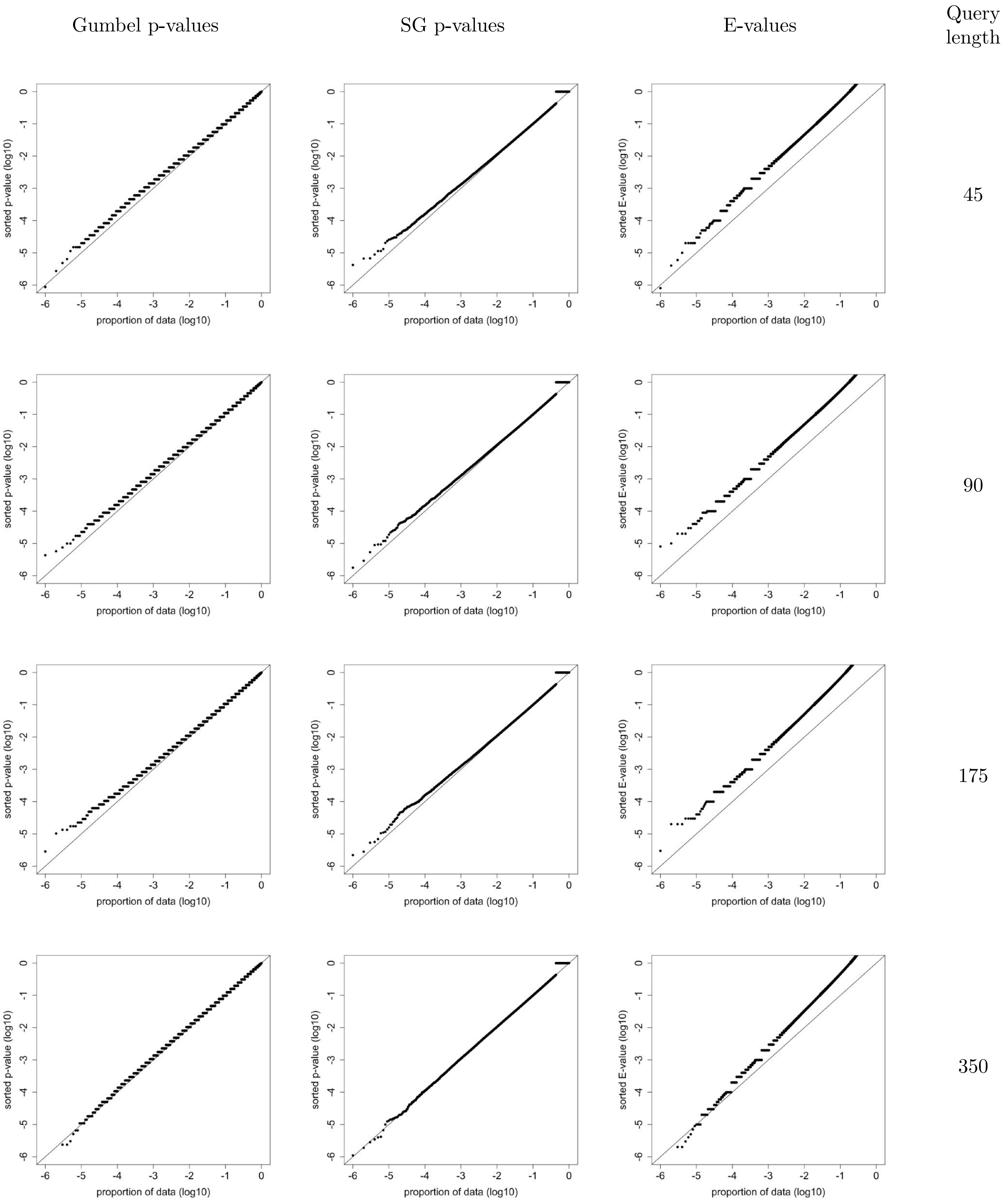

Figure S21: **Using BLAST to search 10^6 shuffled queries against the SCOP database with PAM30 (9,1)**. Same as Figure S6 except searching using PAM30 (9,1).

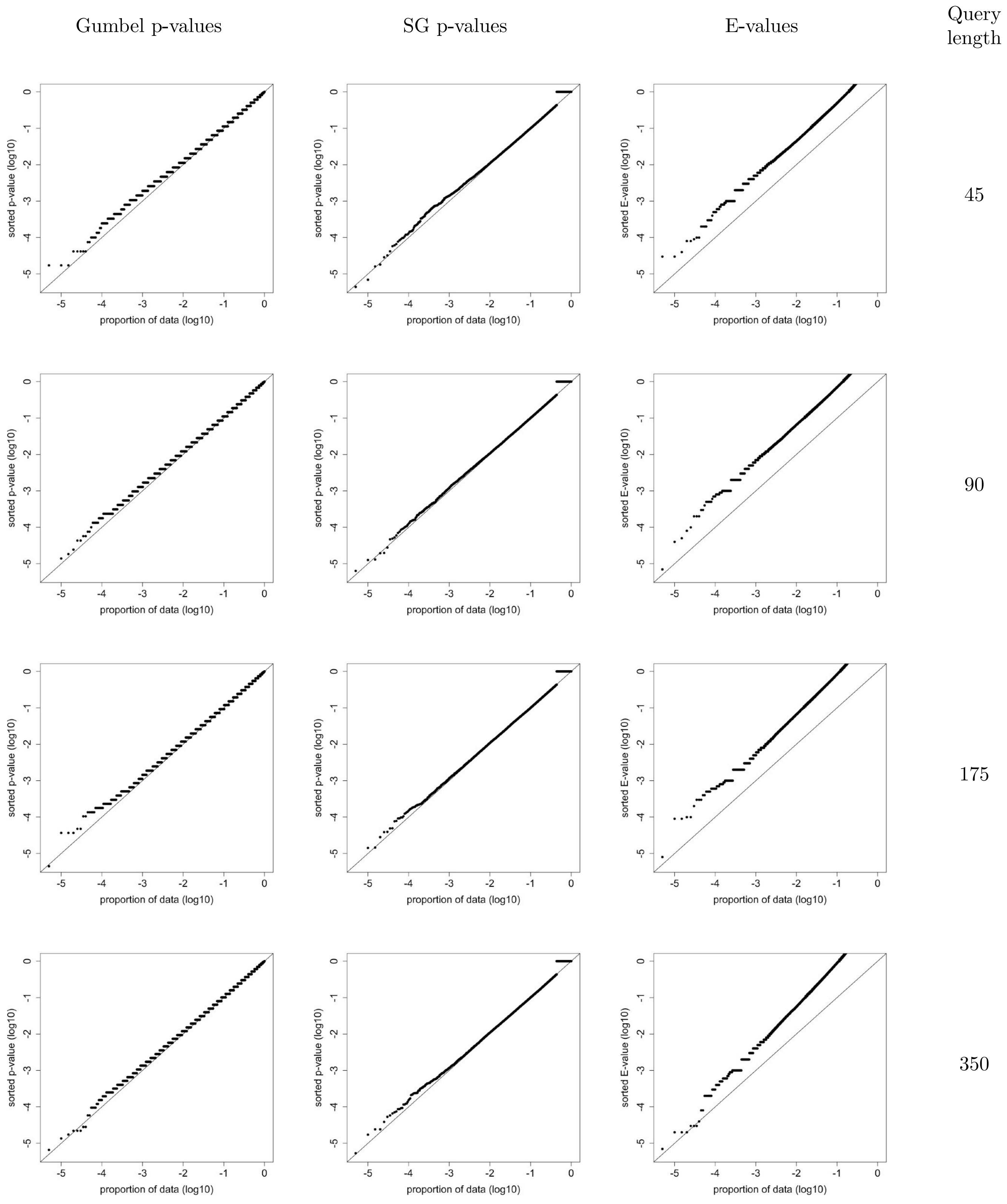

Figure S22: **Using BLAST to search 200,000 shuffled *iid-sampled* queries against the Swiss-Prot database using PAM30 (9,1)**. Same as Figure S7 except using PAM30 (9,1).

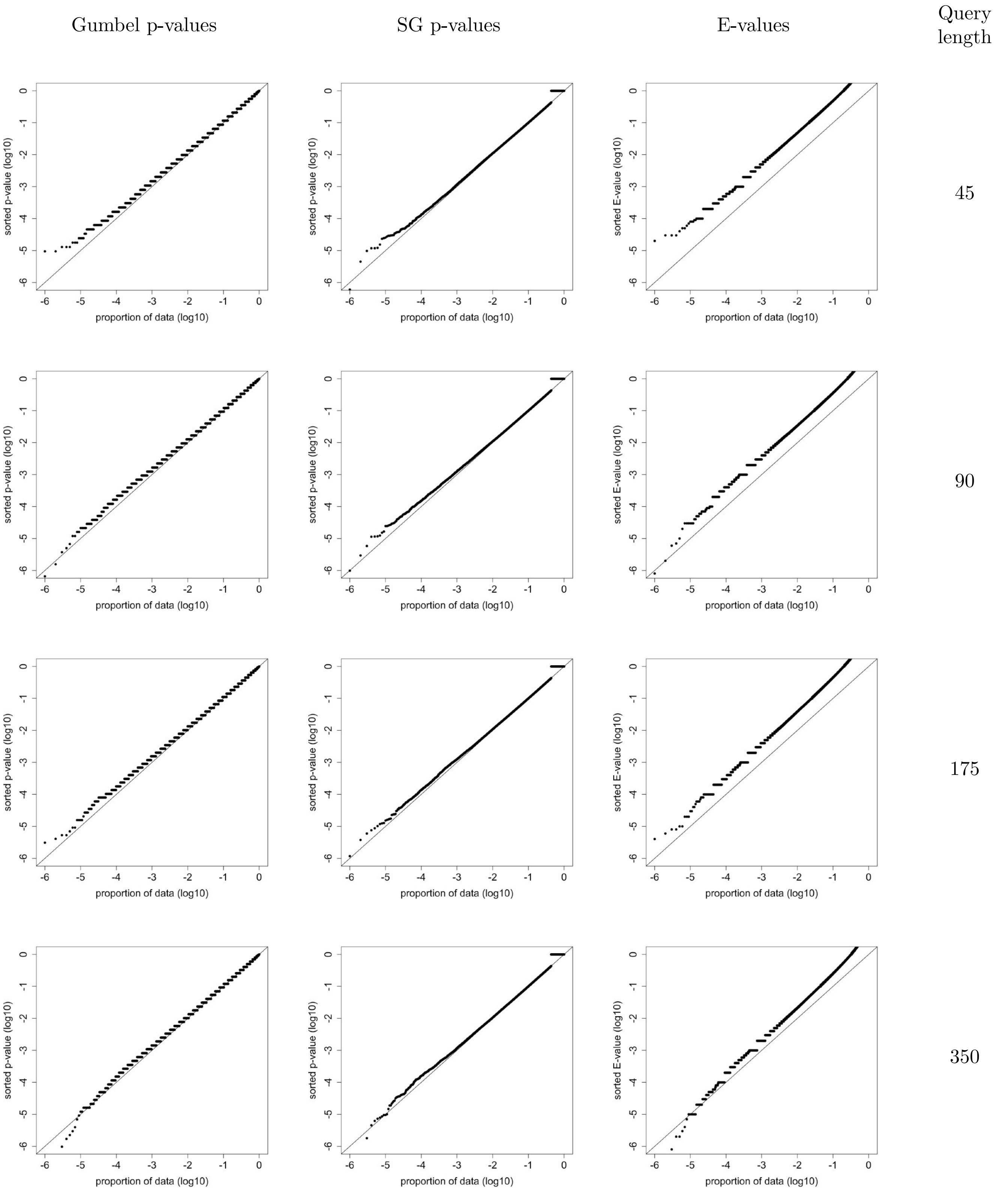

Figure S23: **Using BLAST to search 10^6 shuffled queries against the Swiss-Prot database with PAM70 (10,1).** Same as Figure S5 except searching using PAM70.

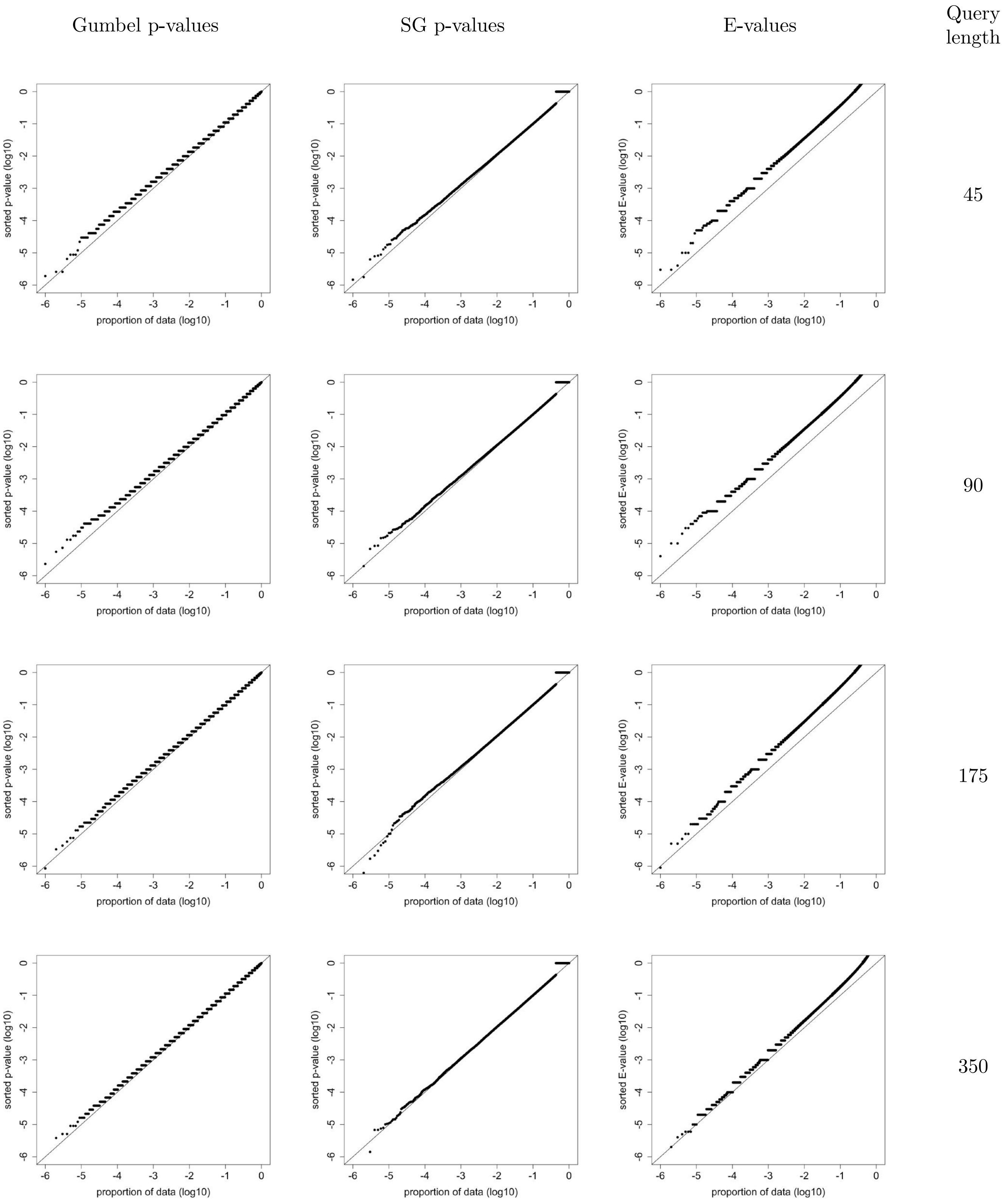

Figure S24: **Using BLAST to search 10^6 shuffled queries against the SCOP database with PAM70 (10,1).** Same as Figure S6 except searching using PAM70.

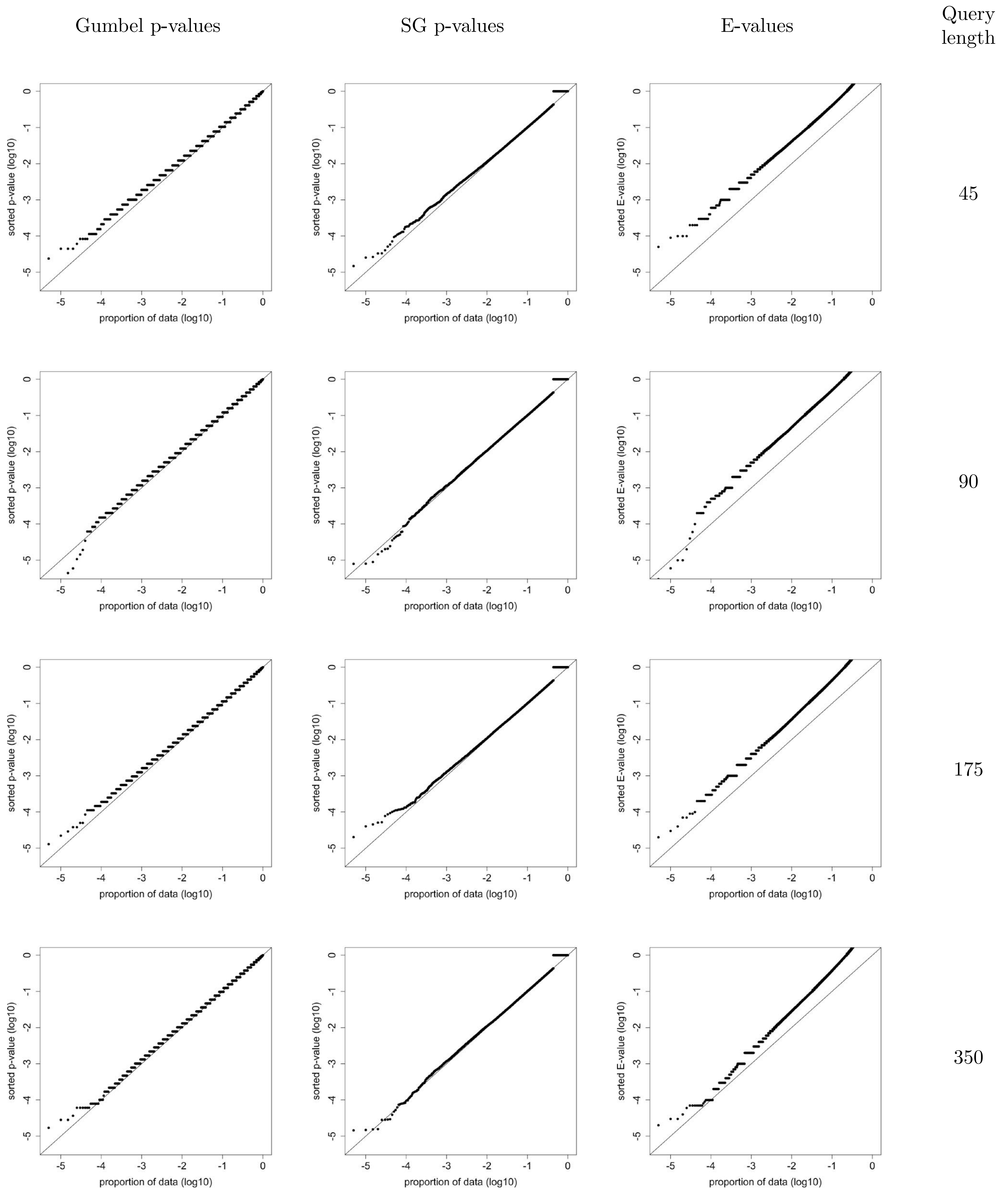

Figure S25: **Using BLAST to search 200,000 shuffled *iid-sampled* queries against the Swiss-Prot database using PAM70 (10,1)**. Same as Figure S7 except using PAM70 (10,1).

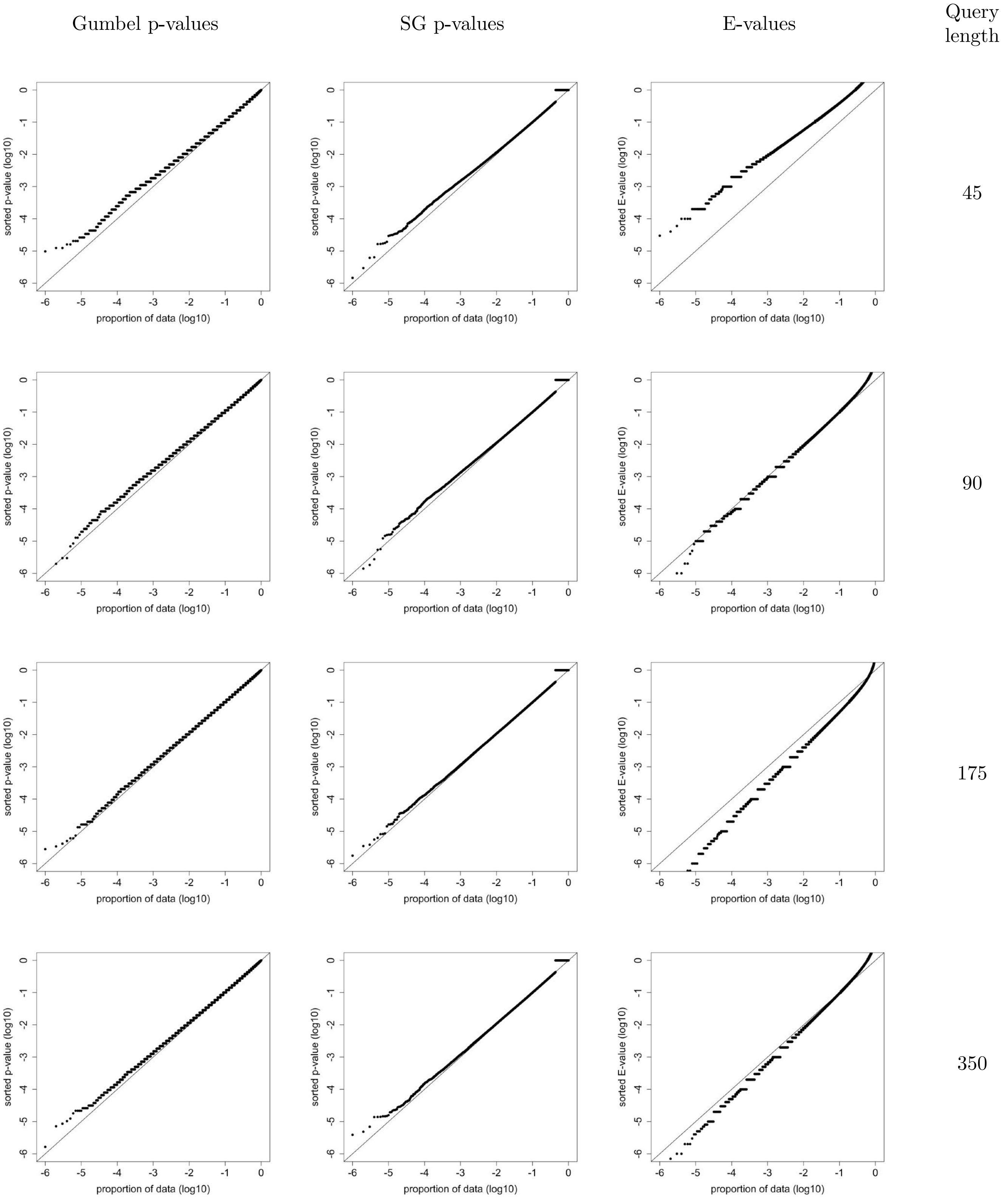

Figure S26: **Using BLAST to search 10^6 shuffled queries against the Swiss-Prot database with PAM250 (14,2)**. Same as Figure S5 except searching using PAM250. Only 7 of the length 45 shuffles have an E-value $\leq 10^{-4}$ instead of the expected 100. At the same time there are 4.2 times more length 175 shuffles with an E-value $\leq 10^{-3}$ than expected (p-value of 0).

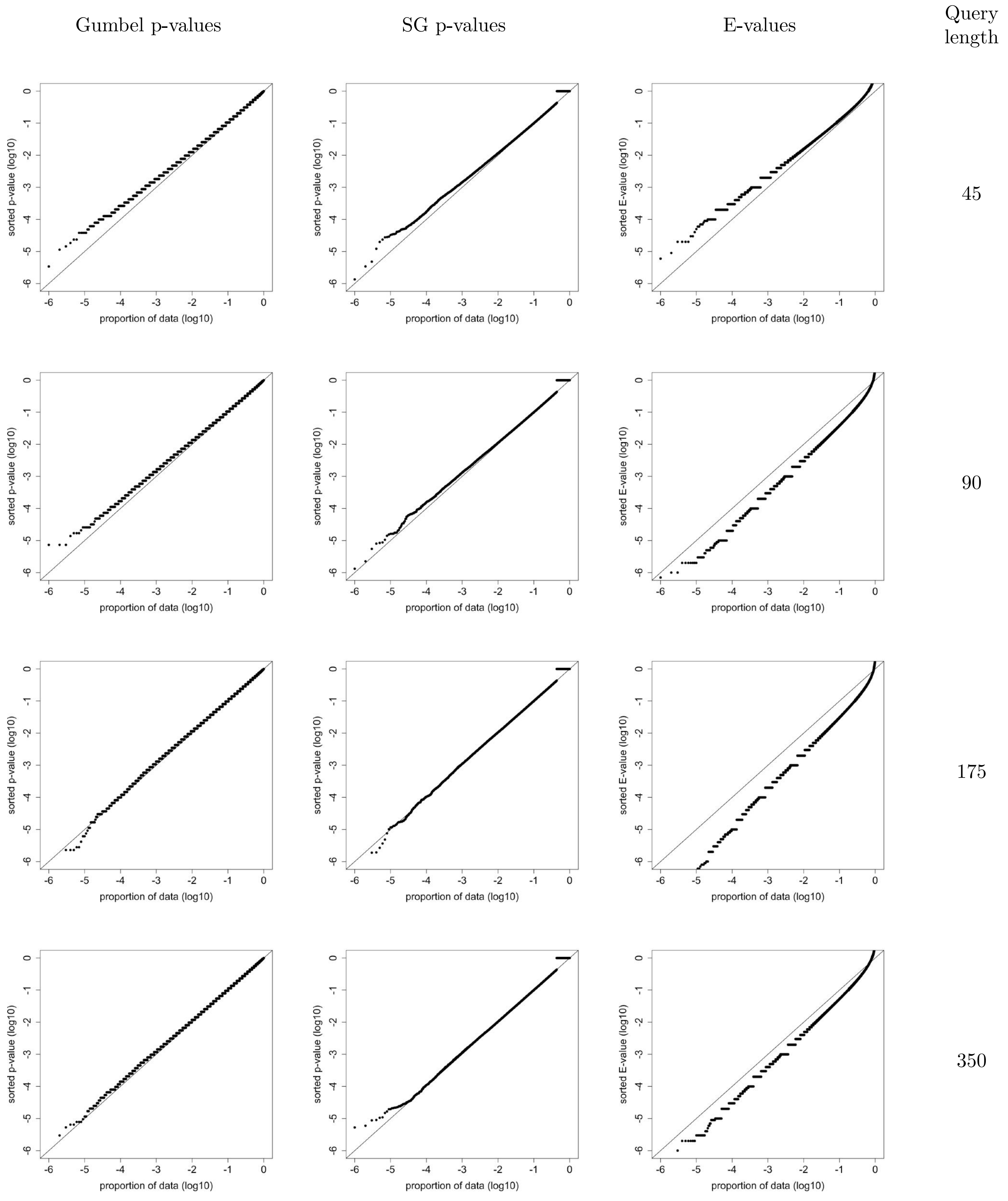

Figure S27: **Using BLAST to search 10^6 shuffled queries against the SCOP database with PAM250 (14,2)**. Same as Figure S6 except searching using PAM250. There are 15.4% of the length 175 shuffles that with an E-value ≤ 0.05 (p-value of 0).

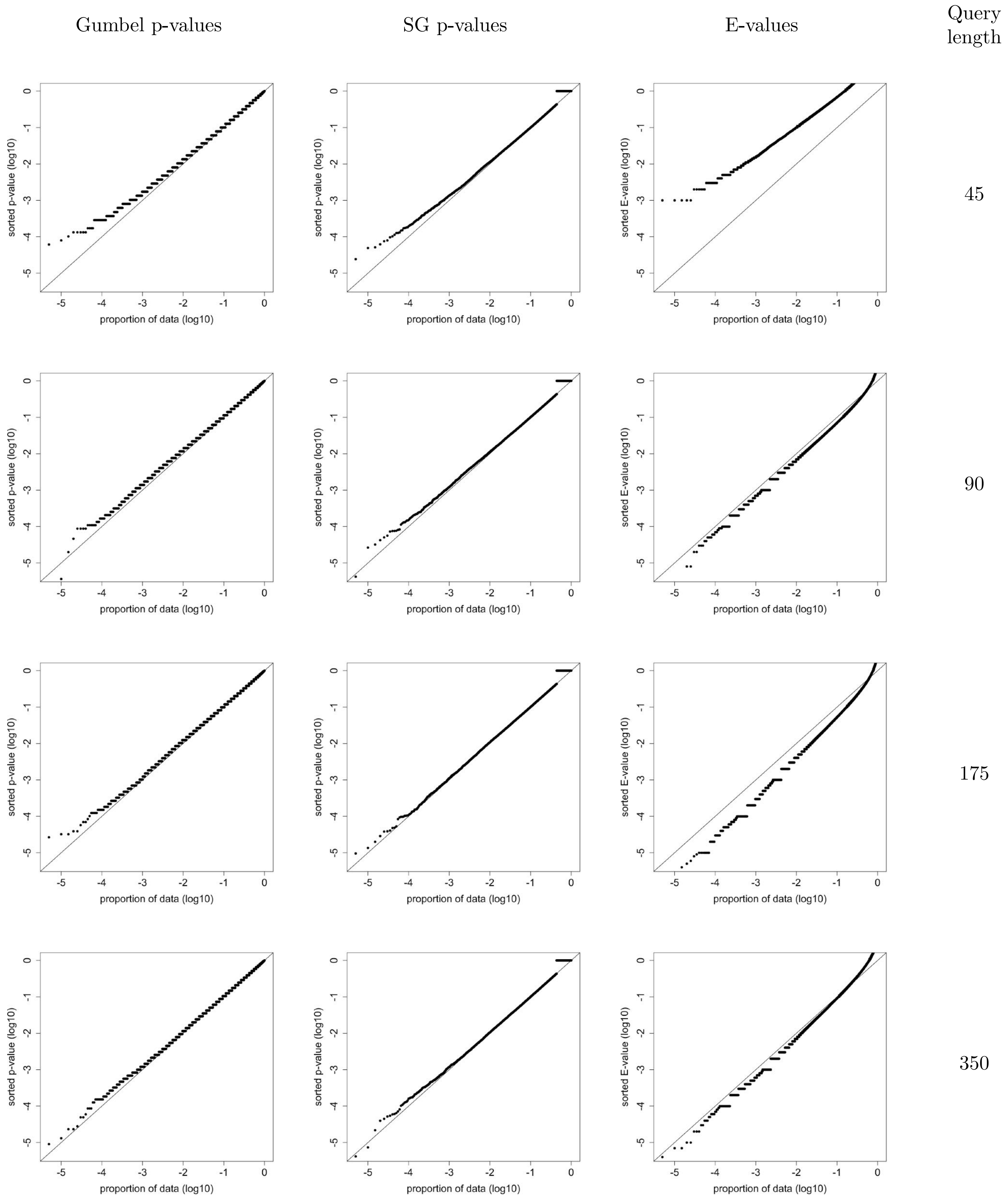

Figure S28: **Using BLAST to search 200,000 shuffled *iid-sampled* queries against the Swiss-Prot database using PAM250 (14,2)**. Same as Figure S7 except using PAM250 (14,2). Only 5 of the length 45 shuffles have an E-value $\leq 10^{-3}$ instead of the expected 200. At the same time there are 4.2 times more length 175 shuffles with an E-value $\leq 10^{-3}$ than expected (p-value of $7 \cdot 10^{-253}$).

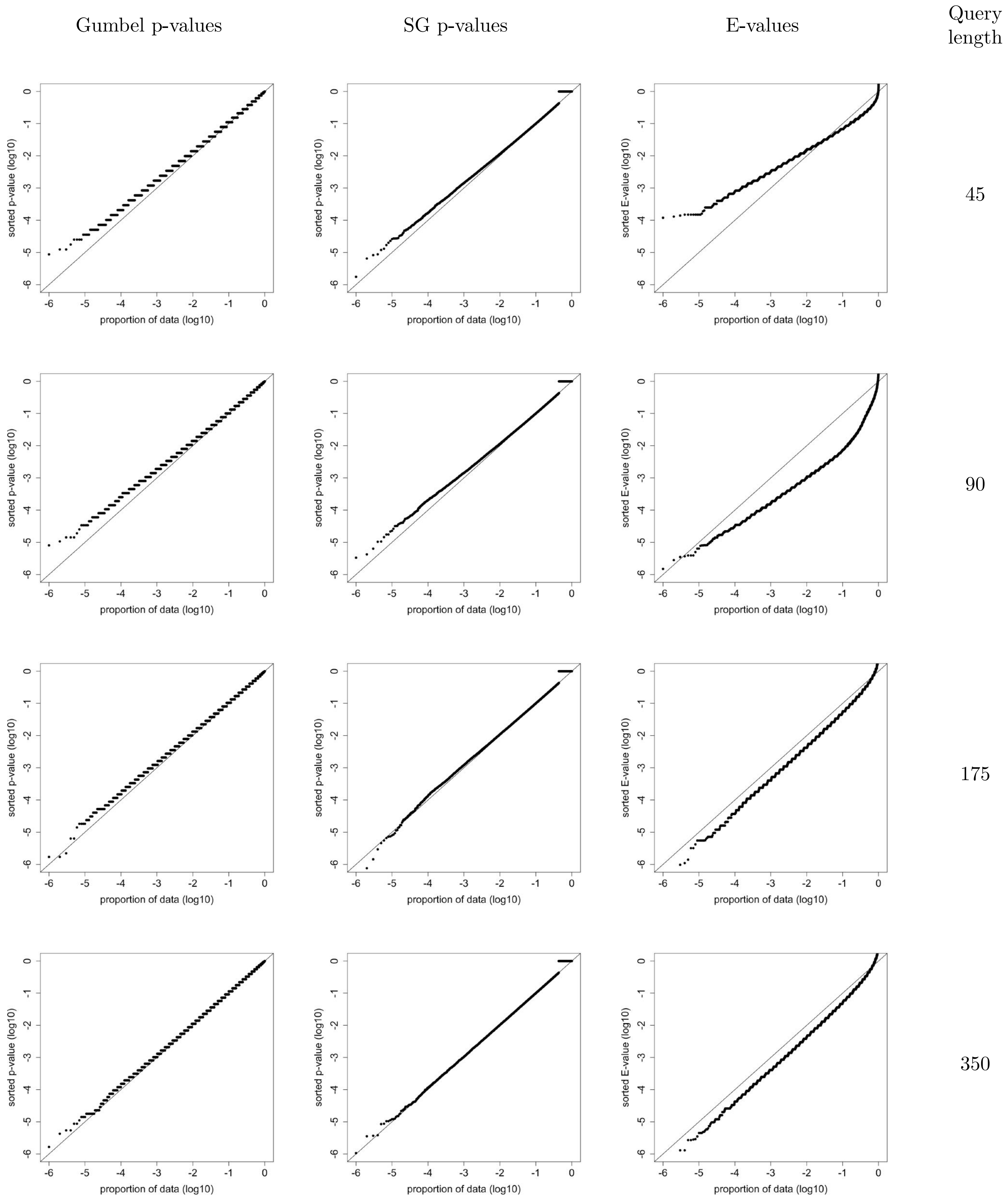

Figure S29: **Using AB-BLAST to search the SCOP database with BLOSUM62 (9,2)**. Similar to figure S6 except the search engine is AB-BLAST using BLOSUM62 with AB-BLAST's default gap penalties of (9,2). Notably AB-BLAST's E-values are not well-calibrated: for queries length of 45 they are at the same time too liberal for some thresholds and overly conservative at others, e.g., 6.1% of the E-values are ≤ 0.05 (binomial test p-value is 0) but none of the 10^6 E-values is $\leq 10^{-4}$, and moreover, 13.9% of the queries of length 90 have an E-values ≤ 0.01 (p-value is 0). The E-values are also clearly too liberal for queries of length 175, e.g., 9.8% of the E-values are ≤ 0.05 (p-value is 0), and 10% of the E-values of 350-long queries are ≤ 0.05 (p-value is 0). At the same time the SG_{50} p-values offer a valid and fairly well calibrated alternative for all query lengths.

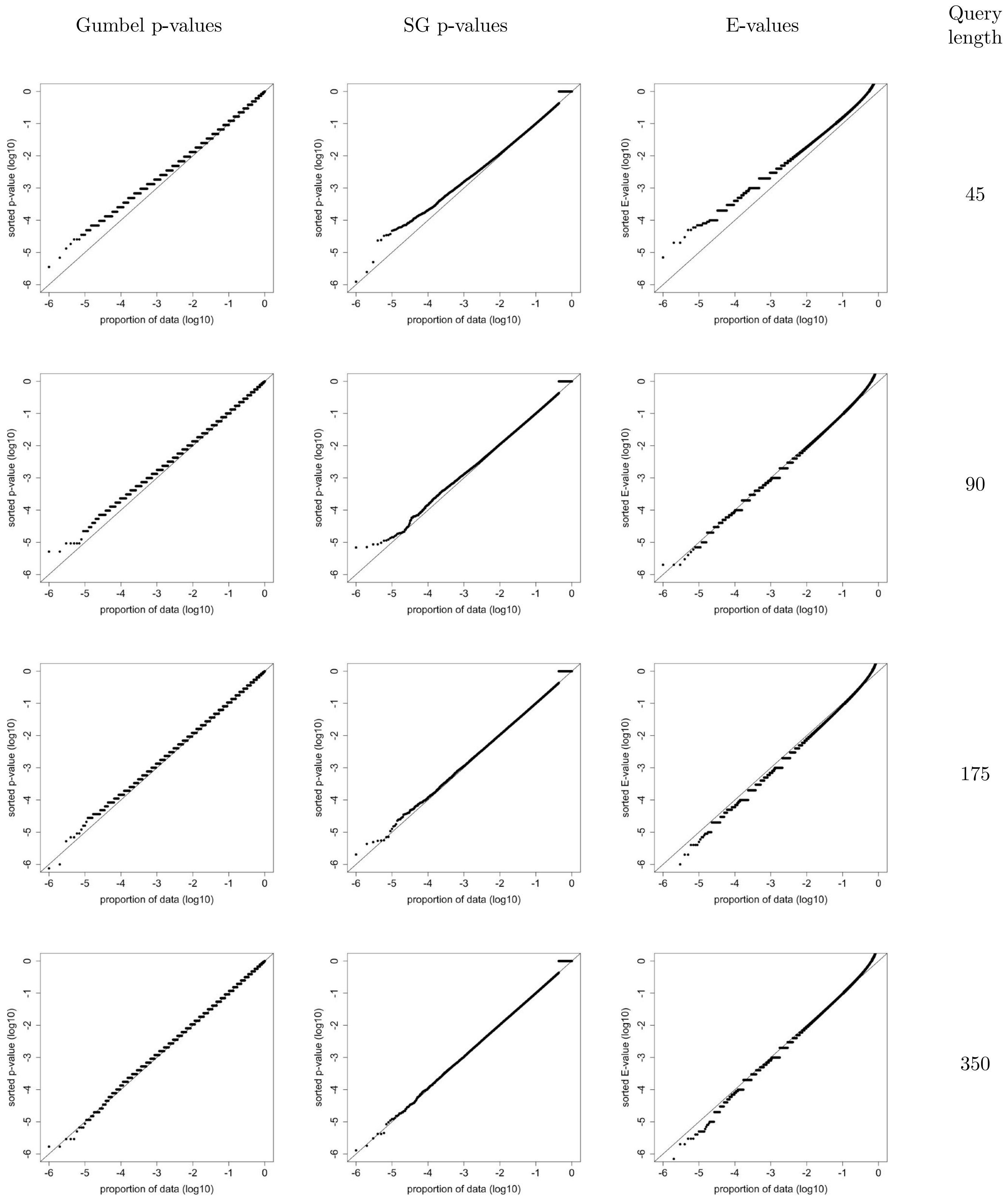

Figure S30: **Using BLAST to search 10^6 shuffled queries against the ASTRAL40 database with BLOSUM62 (11,1)**. Same as Figure S5 except searching ASTRAL40 instead of Swiss-Prot. Notably the BLAST E-values can be clearly too liberal for the queries of lengths 175, e.g., 6.1% of the E-values are ≤ 0.05 (binomial test p-value is 0), and length 350, e.g., 5.4% of the E-values are ≤ 0.05 (p-value is $2.8 \cdot 10^{-87}$). In addition, they are also too liberal for length 90, e.g., 0.18% of the E-values are ≤ 0.001 (p-value is $5 \cdot 10^{-110}$), and for length 700 (not shown), e.g., 0.13% of the E-values are ≤ 0.001 (p-value is $1 \cdot 10^{-17}$). In contrast, the SG_{50} p-values are valid and generally better calibrated for all query lengths.

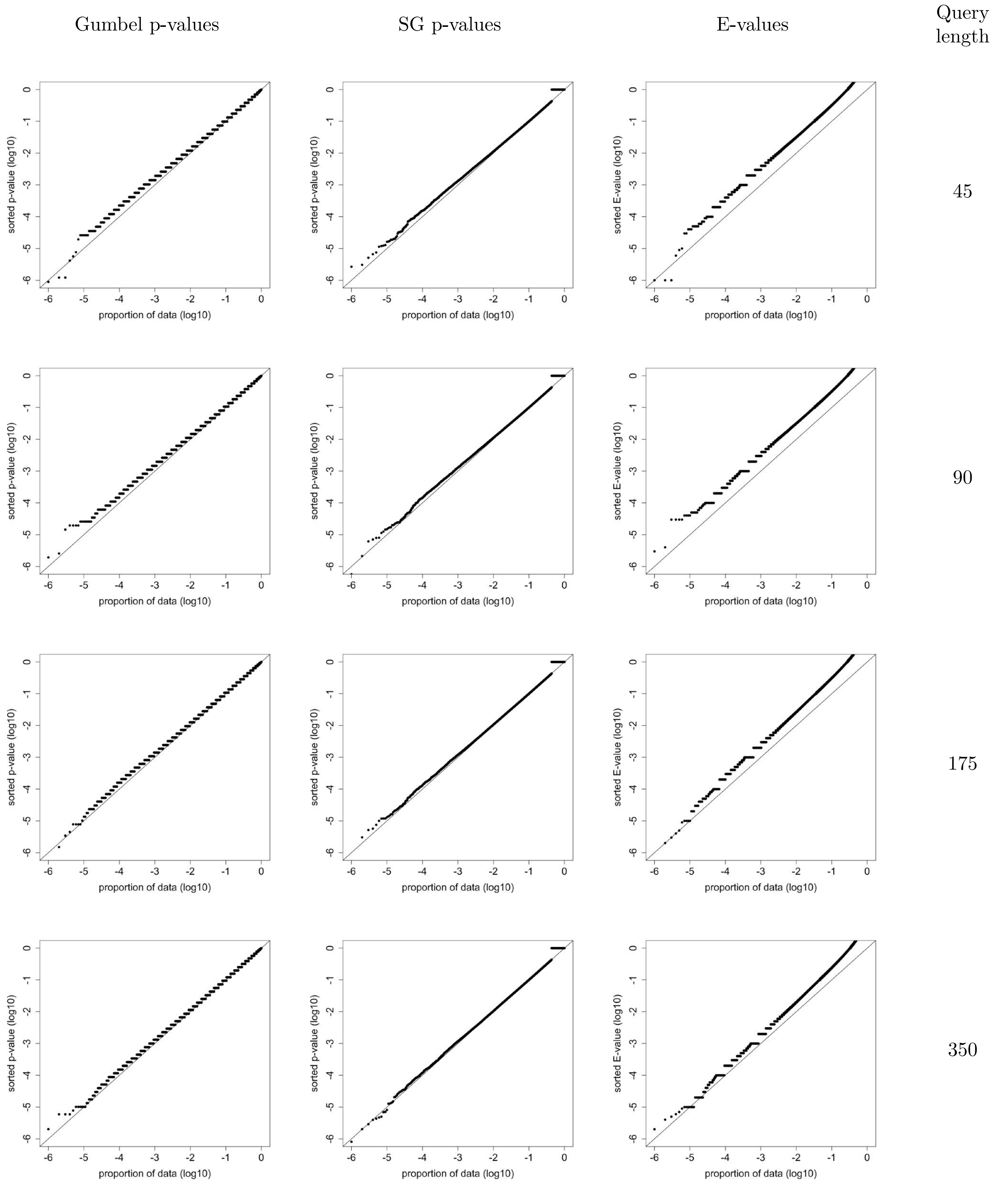

Figure S31: **Using BLAST to search 10^6 shuffled queries against the ASTRAL40 database with PAM70 (10,1)**. Same as Figure S30 except searching using PAM70 (10,1) instead of BLOSUM62 (11,1). In contrast with the latter case, here BLAST's E-values are consistently conservative. While both the E-values and the SG_{50} p-values are valid here, the E-values are significantly more conservative than the p-values.

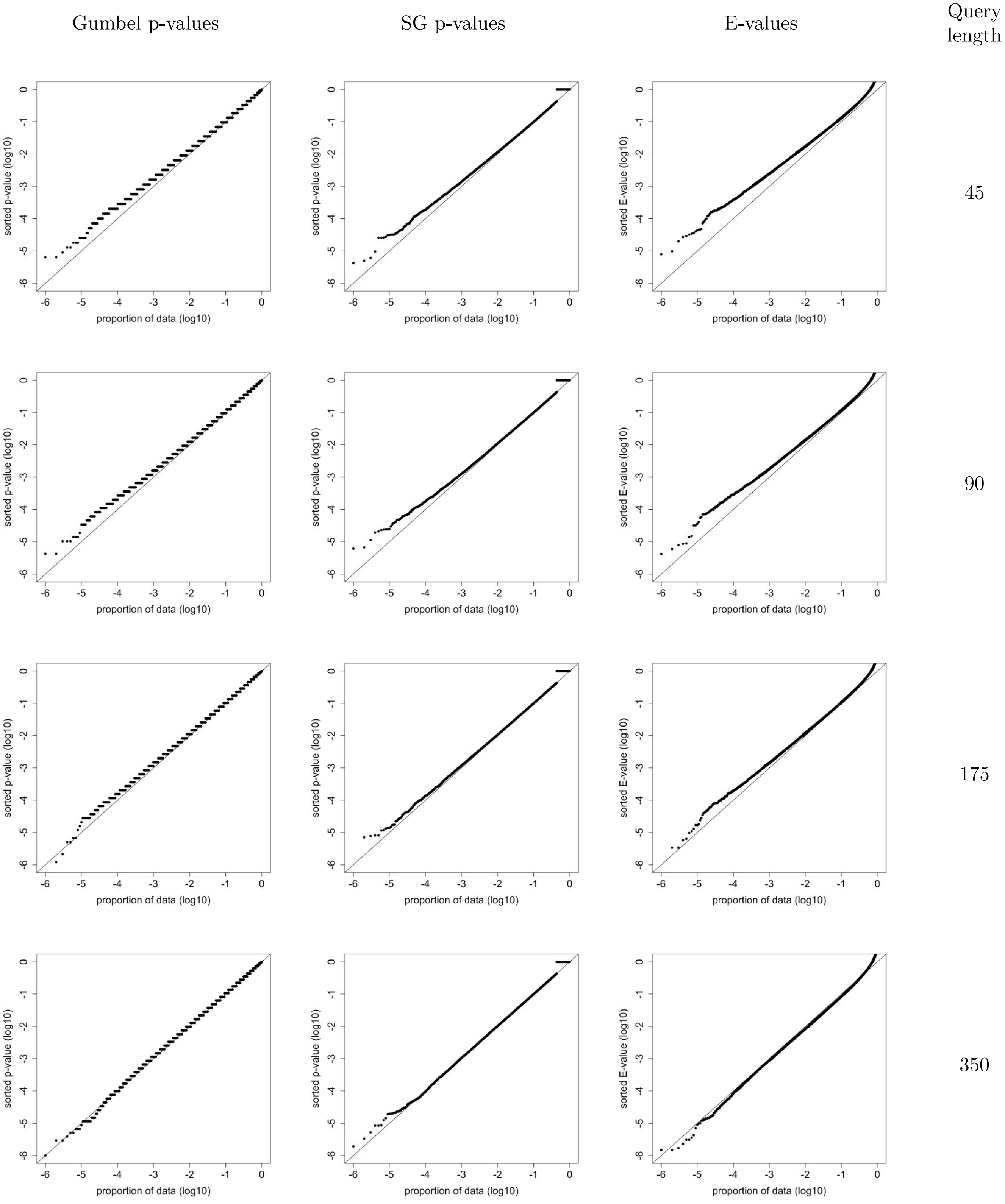

Figure S32: **Using FASTA to search the ASTRAL40 database with BLOSUM62 (11,1)**. Same as Figure S30 except searching with FASTA. While not clearly visible, the FASTA E-values are too liberal for the query of length 350, e.g., 6% of the E-values are ≤ 0.05 (binomial test p-value is 0), whereas the SG_{50} p-values are (a) valid, and (b) better calibrated for all query lengths.

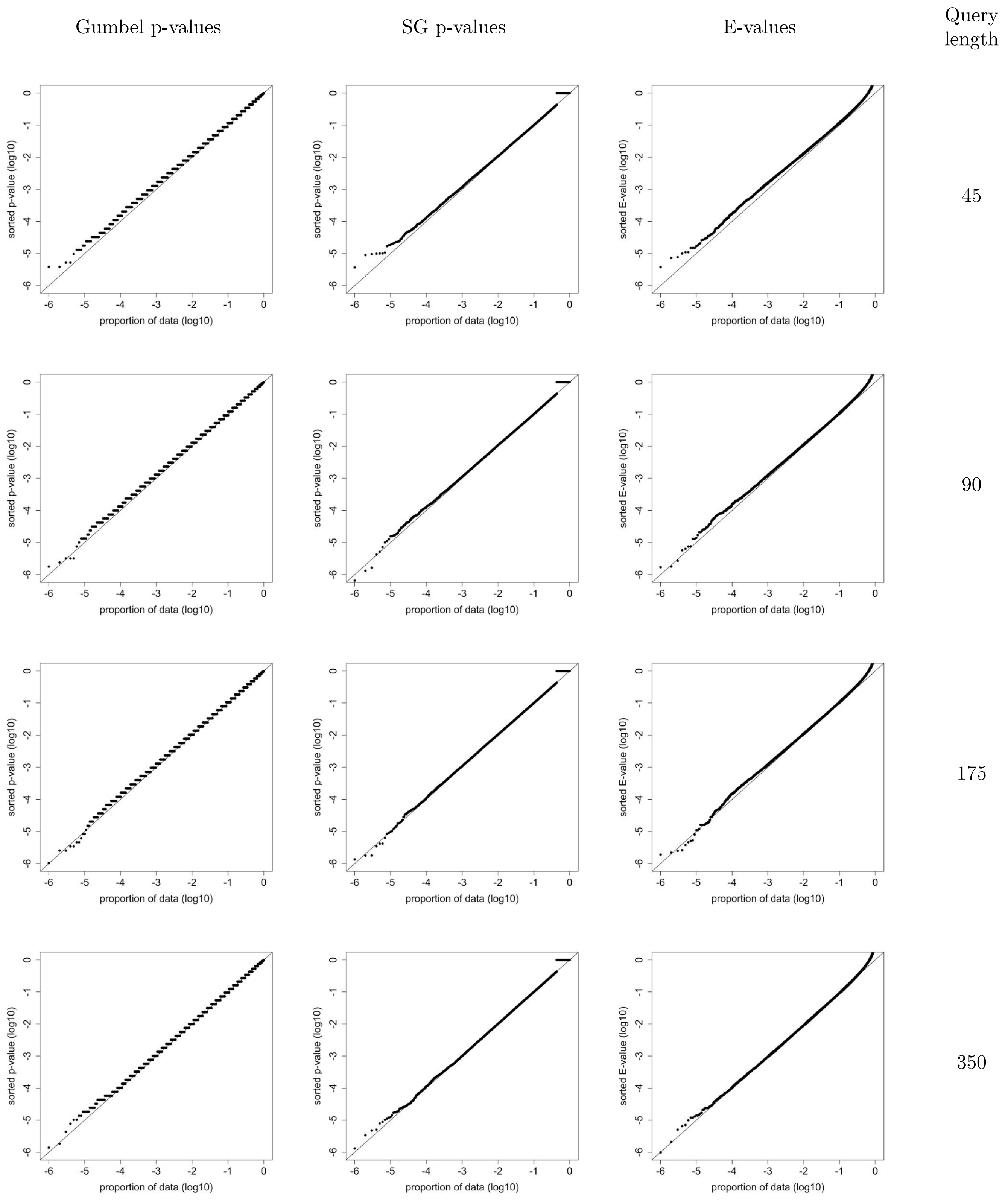

Figure S33: **Using FASTA to search the ASTRAL40 database with PAM70 (10,1)**. Same as Figure S32 except searching with PAM70 (10,1) instead of BLOSUM62 (11,1). Similarly to the latter case, while not clearly visible, the FASTA E-values are too liberal for the query of length 350, e.g., over 5.5% of the E-values are ≤ 0.05 (binomial test p-value is $1.8 \cdot 10^{-121}$) whereas the SG_{50} p-values are (a) valid, and (b) somewhat better calibrated for all query lengths.

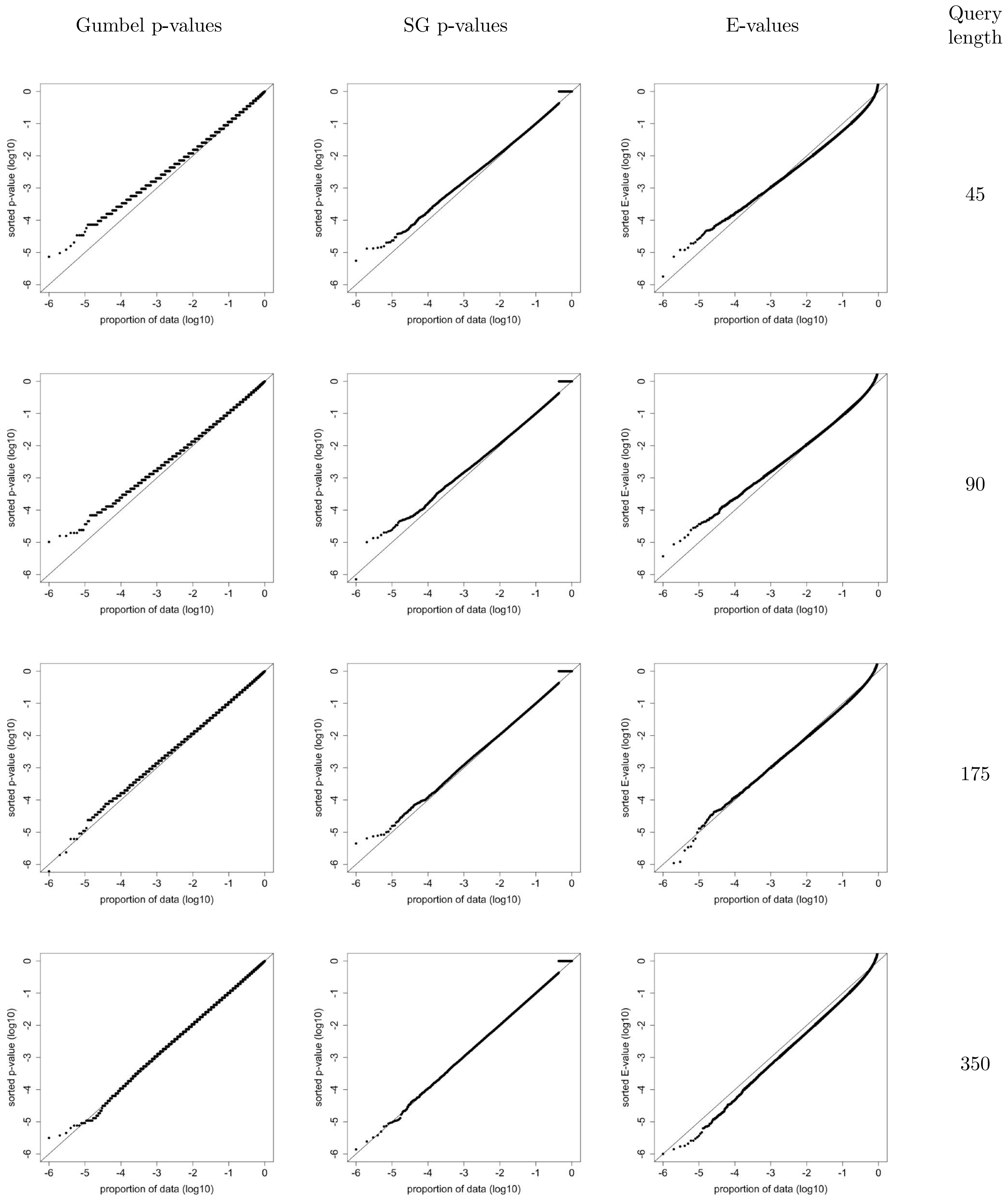

Figure S34: **Using FASTA to search the ASTRAL40 database with PFASUM60 (15,1)**. Same as Figure S32 except searching with PFASUM60 (15,1) instead of BLOSUM62 (11,1). In this case it is clearly visible that FASTA's E-values can be too liberal for length 45, e.g., 8.4% of the E-values are ≤ 0.05 (binomial test p-value is 0), and length 350, e.g., 8.0% of the E-values are ≤ 0.05 (p-value is 0). But they are also too liberal for length 90, e.g., 5.6% of the E-values are ≤ 0.05 (p-value is $1.8 \cdot 10^{-182}$), and length 175, e.g., 6.3% of the E-values are ≤ 0.05 (p-value is 0). In contrast, the SG_{50} p-values are valid and generally better calibrated for all query lengths.

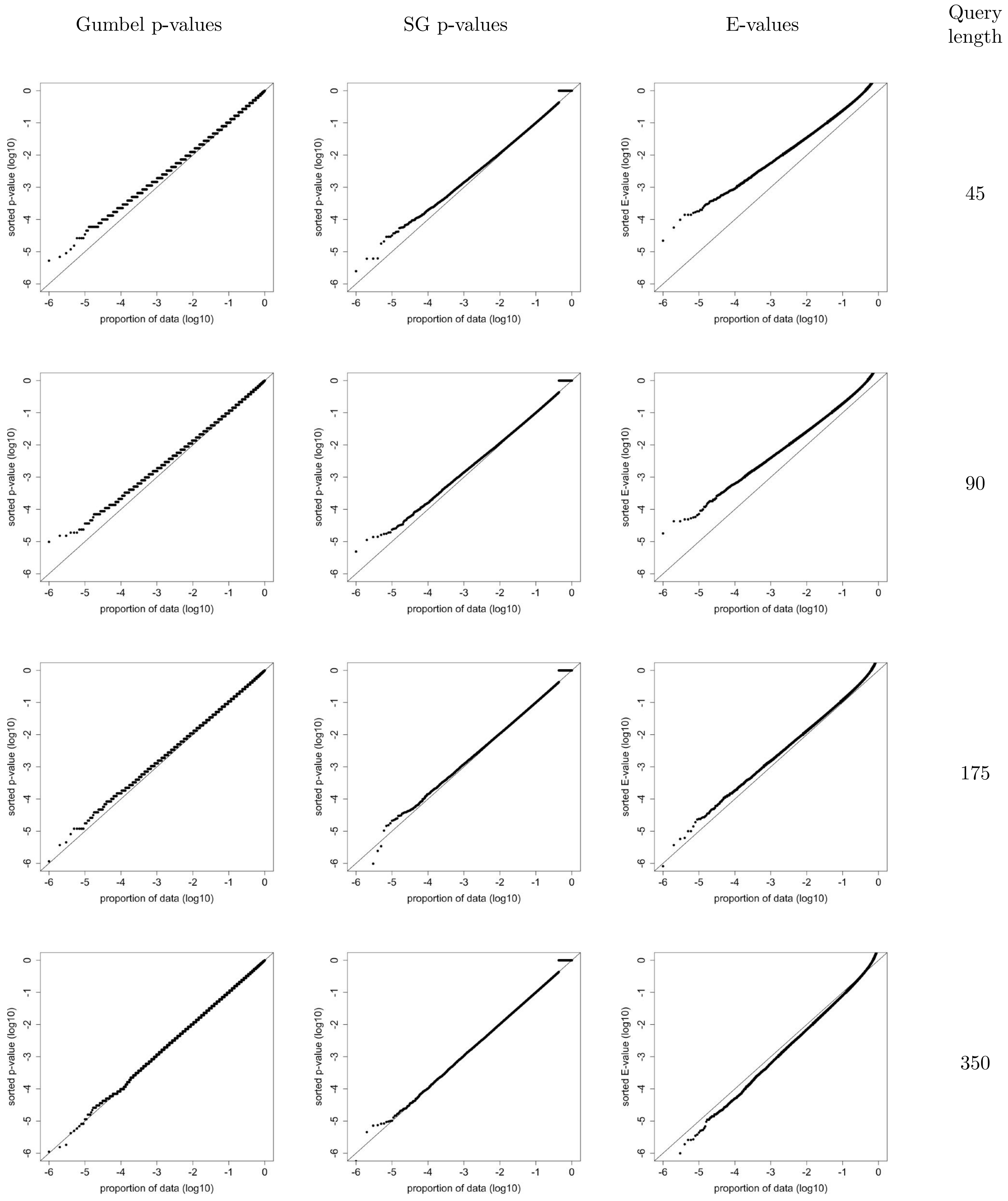

Figure S35: **Using SSEARCH to search the ASTRAL40 database with PFASUM60 (15,1)**. Same as Figure S30 except searching with SSEARCH using PFASUM60 with the (15,1) gap penalties recommended by its authors [15]. Notably the SSEARCH (FASTA) E-values are overly conservative for queries of length 45 they are significantly too liberal for the query of length 350, e.g., 1.5% of the E-values are ≤ 0.01 (binomial test p-value is 0). In contrast, the SG_{50} p-values are valid and better calibrated for all query lengths.

S1.5 The `blastp` wrapper

We implemented Algorithm 1 as part of a wrapper script for **blastp**. Essentially, users can seamlessly invoke **blastp** through the wrapper, resulting in a more reliable statistical estimation in terms of SG-pvalue (Figure S36). The Apache licensed source code is available at (`https://github.com/batmen-lab/SGPvalue`).

Usage:
python blastp_wrapper.py [sgpvalue_options] blastp [blastp_options]

Options:

sgpvalue_options:

–num_shuffle <NUM_SHUFFLE>	The number of null
–lookup_url <LOOKUP_URL>	The SG look-up table
–blastp_url <BLASTP_URL>	The path to the blastp executable file

blastp_options:

-db <DB>	BLAST database name
-query <QUERY>	Query file name
-out <OUT>	Output file name
-outfmt <OUTFMT>	Alignment view options
-matrix <MATRIX>	Scoring matrix name
-evalue <EVALUE>	Expect value (E) for saving hits
-num_descriptions <NUM_DESCRIPTIONS>	Show descriptions for this number of database sequences
-num_alignments <NUM_ALIGNMENTS>	Show alignments for this number of database sequences
-max_target_seqs <MAX_TARGET_SEQS>	Number of aligned sequences to keep
-max_hsps <MAX_HSPS>	Maximum number of HSPs (alignments) to keep
-word_size <WORD_SIZE>	Word size of initial match. Valid word sizes are 2-7
-gapopen <GAPOPEN>	Cost to open a gap
-gapextend <GAPEXTEND>	Cost to extend a gap
-threshold <THRESHOLD>	Minimum score to add a word to the BLAST lookup table
-comp_based_stats <COMP_BASED_STATS>	Use composition-based statistics
-xdrop_gap_final <XDROP_GAP_FINAL>	Heuristic value (in bits) for final gapped alignment
-window_size <WINDOW_SIZE>	Multiple hits window size, use 0 to specify 1-hit algorithm

```
BLASTP 2.14.0+


Reference: Stephen F. Altschul, Thomas L. Madden, Alejandro A.
Schaffer, Jinghui Zhang, Zheng Zhang, Webb Miller, and David J.
Lipman (1997), "Gapped BLAST and PSI-BLAST: a new generation of
protein database search programs", Nucleic Acids Res. 25:3389-3402.


Reference for composition-based statistics: Alejandro A. Schaffer,
L. Aravind, Thomas L. Madden, Sergei Shavirin, John L. Spouge, Yuri
I. Wolf, Eugene V. Koonin, and Stephen F. Altschul (2001),
"Improving the accuracy of PSI-BLAST protein database searches with
composition-based statistics and other refinements", Nucleic Acids
Res. 29:2994-3005.



Database: scop_fa_represeq_lib_latest_all.fasta
           35,644 sequences; 6,930,552 total letters



Query= 8107723_t3_origin

Length=276
                                                                      Score     E
Sequences producing significant alignments:                          (Bits)  Value

8107723 FA=4002088 FA-PDBID=7OIT_AAA FA-UNIID=A0A140TAH5              34.7    0.034
8093645 FA=4002088 FA-PDBID=6QH6_M FA-UNIID=Q3ZC13                    34.3    0.035
8023630 FA=4002088 FA-PDBID=2VGL_M FA-UNIID=P84092                    34.3    0.036
8023950 FA=4002088 FA-PDBID=1H6E_A FA-UNIID=Q96CW1                    33.9    0.051
8021938 FA=4000912 FA-PDBID=1EL0_A FA-UNIID=P22362                    29.1    0.39
8026973 FA=4003124 FA-PDBID=1PYF_A FA-UNIID=P46336                    30.7    0.56
8055168 FA=4007555 FA-PDBID=1WJ3_A FA-UNIID=Q9P232                    28.3    1.5
8099493 FA=4000029 FA-PDBID=6ZZP_A FA-UNIID=Q4FRT2                    28.7    2.3
8082470 FA=4000945 FA-PDBID=4V6U_BM FA-UNIID=Q8U2F9                   28.3    2.5
8051630 FA=4007439 FA-PDBID=5U0P_Q FA-UNIID=P87306                    28.3    3.5
8095272 FA=4003216 FA-PDBID=6OVI_A FA-UNIID=Q5ZYF2                    27.9    3.8
```

A: `blastp`

```
BLASTP 2.14.0+


Reference: Stephen F. Altschul, Thomas L. Madden, Alejandro A.
Schaffer, Jinghui Zhang, Zheng Zhang, Webb Miller, and David J.
Lipman (1997), "Gapped BLAST and PSI-BLAST: a new generation of
protein database search programs", Nucleic Acids Res. 25:3389-3402.


Reference for composition-based statistics: Alejandro A. Schaffer,
L. Aravind, Thomas L. Madden, Sergei Shavirin, John L. Spouge, Yuri
I. Wolf, Eugene V. Koonin, and Stephen F. Altschul (2001),
"Improving the accuracy of PSI-BLAST protein database searches with
composition-based statistics and other refinements", Nucleic Acids
Res. 29:2994-3005.



Database: scop_fa_represeq_lib_latest_all.fasta
           35,644 sequences; 6,930,552 total letters



Query= 8107723_t3_origin

Length=276
                                                                      Score     E    SG
Sequences producing significant alignments:                          (Bits)  Value  p-value

8107723 FA=4002088 FA-PDBID=7OIT_AAA FA-UNIID=A0A140TAH5              34.7    0.034 0.058
8093645 FA=4002088 FA-PDBID=6QH6_M FA-UNIID=Q3ZC13                    34.3    0.035 0.071
8023630 FA=4002088 FA-PDBID=2VGL_M FA-UNIID=P84092                    34.3    0.036 0.071
8023950 FA=4002088 FA-PDBID=1H6E_A FA-UNIID=Q96CW1                    33.9    0.051 0.087
8021938 FA=4000912 FA-PDBID=1EL0_A FA-UNIID=P22362                    29.1    0.39  0.432
8026973 FA=4003124 FA-PDBID=1PYF_A FA-UNIID=P46336                    30.7    0.56  0.417
8055168 FA=4007555 FA-PDBID=1WJ3_A FA-UNIID=Q9P232                    28.3    1.5   0.432
8099493 FA=4000029 FA-PDBID=6ZZP_A FA-UNIID=Q4FRT2                    28.7    2.3   0.432
8082470 FA=4000945 FA-PDBID=4V6U_BM FA-UNIID=Q8U2F9                   28.3    2.5   0.432
8051630 FA=4007439 FA-PDBID=5U0P_Q FA-UNIID=P87306                    28.3    3.5   0.432
```

B: `blastp` **wrapper**

Figure S36: **The search results using `blastp` and the `blastp` wrapper.**

S1.6 The amino acid sequences that were shuffled in the null studies

Table S2: SCOP domain selected for shuffling

```
# SCOP domain selected for shuffling of length=45
>8074028 SF=3000295 SF-PDBID=2JXI_A SF-UNIID=Q88D80
MATTTLGVKLDDPTRERLKAAAQSIDRTPHWLIKQAIFNYLEKLE

# SCOP domain selected for shuffling of length=90
>8107338 SF=3000388 SF-PDBID=7PC8_A SF-UNIID=Q9NSN8
GERTVTIRRQTVGGFGLSIKGGAEHNIPVVVSKISKEQRAELSGLLFIGDAILQINGINV
RKCRHEEVVQVLRNAGEEVTLTVSFLKRAP

# SCOP domain selected for shuffling of length=175
>8107332 SF=3002300 SF-PDBID=7ECQ_A SF-UNIID=Q9D8T0
KYKCGLPQPCPEEHLSFRIVSGAANVIGPKICLEDKMLMSSVKDNVGRGLNIALVNGVSG
ELLEARAFDMWAGDVNDLLKFIRPLHEGTLVFVASYDDPATKMNEETRKLFSELGSRNAK
DLAFRDSWVFVGAKGVQNKSPFEQHMKNSKHTNKYEGWPEALEMEGCIPRRSIAG

# SCOP domain selected for shuffling of length=350
>8098538 SF=3000313 SF-PDBID=3WN6_A SF-UNIID=P17654
GQVLFQGFNWESWKENGGWYNFLMGKVDDIAAAGITHVWLPPPSHSVGEQGYMPGRLYDL
DASKYGNEAQLKSLIEAFHGKGVQVIADIVINHRTAEHKDGRGIYCLFEGGTPDSRLDWG
PHMICRDDPYGDGTGNPDTGADFAAAPDIDHLNKRVQRELIGWLDWLKMDIGFDAWRLDF
AKGYSADMAKIYIDATEPSFAVAEIWTSMANGGDGKPNYDQNAHRQELVNWVDRVGGANS
NATAFDFTTKGILNVAVEGELWRLRGEDGKAPGMIGWWPAKATTFVDNHDTGSTQHLWPF
PSDKVMQGYAYILTHPGNPCIFYDHFFDWGLKEEIERLVSIRNRQGIHPA

# SCOP domain selected for shuffling of length=700
>8076415 SF=3000970 SF-PDBID=5BV9_A SF-UNIID=A8FDC4
SNKERFLTLYHQIKSDANGYFSPEGIPYHSIETLICEAPDYGHMTTSEAYSYWLWLEVLY
GHYTRDWSKLEAAWDNMEKYIIPVNEDGNDEQPHMSAYNPSSPATYASEKPYPDQYPSQL
SGARPAGQDPIDGELKSTYGTNETYLMHWLLDVDNWYKYGNLLNPSHKAAYVNTFQRGQQ
ESVWEAIPHPSQDDKSFGKPNEGFMSLFTKENQVPAAQWRYTNATDADARAIQAIYWAKE
LGYNNSTYLDKAKKMGDFLRYGMYDKYFQTIGSGKQGNPYPGNGKGACHYLMAWYTSWGG
GLGDYANWSWRIGASHCHQGYQNPVAAYALSSDKGGLKPSSATGASDWEKTLKRQLEFYV
WLQSKEGAIAGGATNSWNGDYSAYPAGRSTFYDMAYEDAPVYHDPPSNNWFGMQAWPMER
VAELYYIFVKDGDKTSENVQMAKSCITKWVNYALDYIFIGSRPVSDEEGYFLDDQGRRIL
GGTNATVATTSAPGEFWLPGNIAWSGQPDTWNGFQSATGNPNLTAVTKDPTQDTGVLGSL
VKAFTFFAAATKLETGNYTALGVRAKDAAAQLLEVAWNYNDGVGIVTEEEREDYDRFFKK
EVYFPNGWNGTFGQGNQIPGSSTIPSDPQRGGNGVYTSFADLRPNIKQDPAWSSLESKYQ
SSFNEATGKWENGAPVFTYHRFWSQVDMATAYAEYHRLIN
```

Table S3: IID sampled sequences subject to BLAST's amino acid marginal probabilities

we sampled the sequence for a given length from the following marginal distribution
['A', 'R', 'N', 'D', 'C', 'Q', 'E', 'G', 'H', 'I', 'L', 'K', 'M', 'F', 'P', 'S', 'T', 'W', 'Y', 'V']
[0.074, 0.052, 0.045, 0.054, 0.025, 0.034, 0.054, 0.074, 0.026, 0.068, 0.099, 0.058, 0.025, 0.047, 0.039, 0.057, 0.051, 0.013, 0.032, 0.073]

example iid sequence of length=45
>shuffled1_query_len45
QGYFDRIVNNPFTRLDQTFNSMNNCVDRYQPAAPLSMSPENSDTG

example iid sequence of length=90
>shuffled83_query_len90
QESEVMRLAGLSTNSVEDKADKLVNFKKSSGQAERTSPATQWYFKFFKEEKSSGTIFKVA
EQIERNVALSIMLIPEIKPAFVPGTNSMVY

example iid sequence of length=175
>shuffled1_query_len175
CKIGILDLEFNERHAADDQEWIPEKDDDLEVDGVNIATVEHTKKACEDLKRNNGLREEWL
EPETPKRLRALNATKYSSADTRKELRASNSIDDWFQERGATWVKVLAYCVMVGSRGGSAV
QTTVRRLTNIQSMKKAIAMDADALFSDSFKKDGAPEQVPGKQIIASQVLATITES

example iid sequence of length=350
>shuffled1_query_len350
AHVLTHQGQGEVVQHKQHNKEGSQFKPALGRPLVASFELKIWPSDLLGKVLSRATLFTVY
LDRNTRNNSSGKESIVSALENRTPMSAYNVNPVVTQMATGVRPDLIRLNYGSELSGRYLQ
IRRAVSAIYNCRNICRSQKLRARHGYNKAKFIAQVTRFTFEWFQDFQQTTRCALEFIQCE
DNDSDGAFQWEDEDAEYATHLIKGRGCKWDHSPVFGIPGGLSIYQMRLWTISLIAPGLID
TNEDFRWVKFLLTARQNEGHGKIRQFPSYSTFASETDLSLNDDQSEIYTWRVDESSAVYP
VSLFCVQRSAVMQFNTFRPVLPSINNPNYMSQTFHTLYSDLSELILVHKY

example iid sequence of length=700
>shuffled1_query_len700
ILLNVMLEGWSLAAGKKTASTPSSTVLLVQTDHDVKTHKERATLFEIGSKSASRPAVYIK
ATRWFEPADRNRTSSKAMLMVIDFKTFSKQDLDIRFRSNIMKLRFMAHTEIAWGIQWIWR
HLGQAVYFESDHSMMPPGYMLFLQTNVVELDQEPDEEGYTRLKTILANNLAQIRYENAIE
DSIIAARPRETCPSASGLSKDTLRATPFTGSDQTASENFYIKSYSDEGLDFAKLFLNTSS
LRDANAGTLFEIVALFLELELLYPKNMAVLEILPRYVKQGGAYSAGTIAGVLLDGIDETE
ASTRLYLKPLKKFIALRIGKSDIKVDVALKHESEYPFAYPVYSLINRIFTALAGQCEGDT
YANTQRYPLLGPAMGPIIVFCMAGPPVTCRAGNNALPSHETVINGKTSRLQVIDMSSSIR
QGRQPGVSRQASVLGDGIATAKMAKVVDEGDANITVRNADPLRGMNLGDKRSHSMLKGMS
LQHETGPMTLGFKMKALAAFLLAYPAYPWVNTTDRVQAIIFETKSNSQGGPNKGNRSNFF
DRAKLLSAAVAPVSGRCSKFMTMAYTMEDSGTNRLYNSLGVKVLMDMIPHSREQVYMVAM
EYTAAYLRNGQVDNYADGTGHIDQNNVEGLDEHIRSTPGRISLQFVSDRSSPGPRVRELS
ESPPYSLEHLGKTAAMDEKNQSVMEQPLRYQREGIKVVLE
